# Comparative Risk Assessment of Three Native *Heliotropium* Species in Israel

**DOI:** 10.3390/molecules26030689

**Published:** 2021-01-28

**Authors:** Jakob A. Shimshoni, Shimon Barel, Patrick P. J. Mulder

**Affiliations:** 1 Department of Food Quality & Safety, Institute for Postharvest and Food Sciences, Agricultural Research Organization, Volcani Center, Rishon LeTsyion 753593, Israel; 2 Department of Toxicology, Kimron Veterinary Institute, Bet Dagan 50250, Israel; Shimonba@moag.gov.il; 3 Wageningen Food Safety Research, Wageningen University & Research, P.O. Box 230, 6700 AE Wageningen, The Netherlands; patrick.mulder@wur.nl

**Keywords:** *Heliotropium europaeum*, suaveolens, rotundifolium, pyrrolizidine alkaloids, interim relative potency factor

## Abstract

Pyrrolizidine alkaloids (PAs) are genotoxic carcinogenic phytotoxins mostly prevalent in the Boraginaceae, Asteraceae and Fabaceae families. *Heliotropium* species (Boraginaceae) are PA-producing weeds, widely distributed in the Mediterranean region, that have been implicated with lethal intoxications in livestock and humans. In Israel, *H. europaeum*, *H. rotundifolium* and *H. suaveolens* are the most prevalent species. The toxicity of PA-producing plants depends on the PA concentration and composition. PAs occur in plants as mixtures of dozens of various PA congeners. Hence, the risk arising from simultaneous exposure to different congeners has to be evaluated. The comparative risk evaluation of the three *Heliotropium* species was based on recently proposed interim relative potency (iREP) factors, which take into account certain structural features as well as in vitro and in vivo toxicity data obtained for several PAs of different classes. The aim of the present study was to determine the PA profile of the major organ parts of *H. europaeum*, *H. rotundifolium* and *H. suaveolens* in order to assess the plants’ relative toxic potential by utilizing the iREP concept. In total, 31 different PAs were found, among which 20 PAs were described for the first time for *H. rotundifolium* and *H. suaveolens*. The most prominent PAs were heliotrine-*N*-oxide, europine-*N*-oxide and lasiocarpine-*N*-oxide. Europine-*N*-oxide displayed significant differences among the three species. The PA levels ranged between 0.5 and 5% of the dry weight. The flowers of the three species were rich in PAs, while the PA content in the root and flowers of *H. europaeum* was higher than that of the other species. *H. europaeum* was found to pose a higher risk to mammals than *H. rotundifolium*, whereas no differences were found between *H. europaeum* and *H. suaveolens* as well as *H. suaveolens* and *H. rotundifolium*.

## 1. Introduction

**Figure 1:**
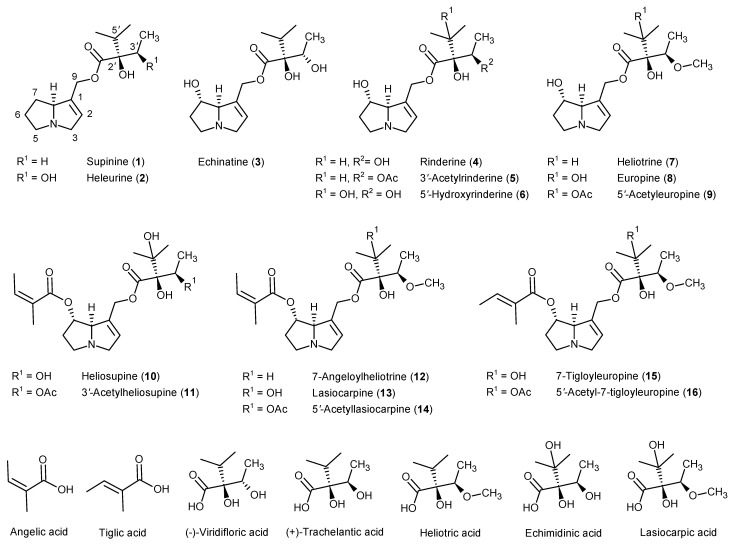
Pyrrolizidine alkaloids (PAs) and corresponding necic acids commonly found in *Heliotropium* species. The corresponding PA-*N*-oxide forms are not shown.

Pyrrolizidine alkaloids (PAs) are secondary plant metabolites that function as chemical defense compounds against various herbivores [1,2,3]. They are highly prevalent in the plant families of Boraginaceae, Asteraceae (*Senecio* and *Eupatorieae* tribes) and Fabaceae (*Crotalarieae* tribe). More than 660 PA and PA-*N*-oxides have been identified and these have been estimated to occur in over 6000 plants [1,2,3]. PAs are composed of a saturated or 1,2-unsaturated necine base esterified with one or two necic acids [1,2]. Common PA types occurring in the *Heliotropium* genus are depicted in Figure 1 [3]. In general, PAs occur in two forms, namely, as the free tertiary base and their corresponding *N*-oxide.

Many PAs exhibit genotoxic effects in humans and farm animals targeting mainly the liver [4,5,6,7]. Most 1,2-dehydro PAs are pro-toxins, requiring metabolic activation by cytochrome P450 to generate a pyrrolic ester as the reactive electrophilic intermediate that subsequently reacts with proteins and nucleic acids [6,7]. Since, upon ingestion, PA-*N*-oxides are reduced in the gut/liver to their corresponding free bases, both PA forms are considered to be toxic [2,7,8]. The toxicity of PA-producing plants mostly depends on the absolute PA concentration but also on the plants’ PA composition, as the toxic potential of individual PAs and their *N*-oxides widely differs [4,7]. The structural features determining the toxic potencies of PAs have been extensively studied and reviewed [2,4,9]. 

PA-containing plants commonly grow in pastures and agricultural fields, resulting in occasional cross-contamination of crops and agricultural produce [1,3,4]. As PAs occur as mixtures of dozens of various PA congeners, the risk arising from simultaneous exposure to different congeners has to be evaluated [9,10]. Although the genotoxic potency of different congeners may vary substantially by several orders of magnitude, the current approach to assess the risk of PA mixtures in a sample is to assign to all of them the same potency as the most toxic congeners (namely, lasiocarpine or riddelliine), resulting in a clear risk over-estimation [9,10]. 

Recently, Merz and Schrenk suggested assigning interim relative potency (iREP) factors to PA congeners of certain structural features [9]. In brief, this approach is based on structural considerations together with acute toxicity data from rodents, cytotoxicity data in mammalian cell cultures and genotoxicity data in Drosophila, obtained for a limited number of PAs. The iREP factors range from 1.0 for the most toxic PAs to 0.01 for the least toxic congeners [9]. The limited available in vivo and in vitro toxicity data indicate that cyclic PA di-esters and 7S-open-chained PA di-esters (compounds **10**–**16** in Figure 1) are markedly more toxic than 7R-open-chained di-esters followed by 7S PA mono-esters (compounds **1**–**9**) [3,9,10]. 

*Heliotropium* species are PA-producing weeds, widely distributed in the Mediterranean region, that have been implicated with lethal PA intoxications in livestock and humans [11,12,13,14,15,16,17,18]. In Israel, 14 different *Heliotropium* species have been reported, with *H. europaeum*, *H. rotundifolium* and *H. suaveolens* being frequently found in pastures [19,20,21]. During February 2014, a herd of 73 replacement mixed breed beef cattle (15–18 months old) from the Galilee region of Israel were fed for six weeks a total mixed ration containing hay contaminated with 15% *Heliotropium europaeum*, resulting in a mortality rate of 33% over a period of 63 days [17]. In nearby agricultural fields, used primarily for hay production, two additional *Heliotropium* species, namely, *H. suaveolens* and *H. rotundifolium*, have been found in high abundance. The total PA content and composition might vary among different *Heliotropium* species as well as being affected by external factors such as climate, geographic location, season and plant organ [4,22,23,24,25]. 

The aims of the present study were: (1) to investigate the PA composition and concentration in the major organ parts (root, stem, leaf and inflorescence) of the most abundant *Heliotropium* species (*H. europaeum*, *H. rotundifolium* and *H. suaveolens*), by liquid chromatography coupled to tandem mass spectrometry (LC-MS/MS) analysis and (2) to perform a comparative risk assessment of the three *Heliotropium* species based on the novel iREP factor concept, in order to classify each species in terms of potential hazard to farm animals and humans.

## 2. Results and Discussion

Pyrrolizidine alkaloids are not only found throughout the plant kingdom in a staggering variety of chemical structures, but they may also occur in a single plant in a highly complex composition. For instance, *Senecio jacobaea* has been reported to produce at least 37 different PAs and the number of identified PAs in *S. pterophorus* even exceeds 50 analogues [26,27]. The development of highly sensitive mass spectrometric techniques such as LC-MS/MS, which allow the simultaneous measurement of free base PAs and PA-*N*-oxides, has greatly facilitated the exploration of plant PA profiles. In our study on the poisoning of cattle fed with feed contaminated with high amounts of *H. europaeum*, we could show the presence of at least 30 PAs (including 15 PA-*N*-oxide forms) [17]. The increased availability of PA reference standards (18 were available for this study) has greatly facilitated the identification and quantification of the major metabolites present in the three species and assisted in the tentative identification of those metabolites for which no analytical standard was available. 

### 2.1. PAs Identified in H. europaeum, H. rotundifolium and H. suaveolens 

*Heliotropium europaeum, H. rotundifolium* and *H. suaveolens* belong to the “Old World” clade within the *Heliotropium* genus [28]. Other important members of this clade, which also occur in Israel, are *H. bovei*, *H. hirsutissimum,* and *H supinum.* Based on their close phylogenetic relationship, their PA profiles may be expected to be rather similar. A comprehensive PA profile of the Boraginaceae family was recently reviewed by El-Shazly and Wink, summarizing the available information on the present status of PAs regarding the structure, distribution, chemistry, chemotaxonomic significance and biological properties [3]. El-Shazly and Wink reported, for *H. europaeum*, europine, acetyleuropine, heleurine, heliotrine, 7-angeloylheliotrine, lasiocarpine, 5′-acetyllasiocarpine and supinine as constituents [3]. For *H. rotundifolium*, europine, 5′-acetyleuropine, heliotrine and lasiocarpine, and for *H. suaveolens*, echinatine, rinderine, heliotrine and lasiocarpine have been reported [3]. *H. bovei* reportedly contains europine, 7-acetyleuropine, lasiocarpine and 5′-acetyllasiocarpine; *H. hirsutissimum* contains europine, heliotrine, heleurine, lasiocarpine, 3′-acetyllasiocarpine, 5′-acetyllasiocarpine and supinine; and *H. supinium* contains echinatine, heliosupine, heliotrine, 7-angeloylheliotridine, lasiocarpine and supinine [3]. 

**Figure 2:**
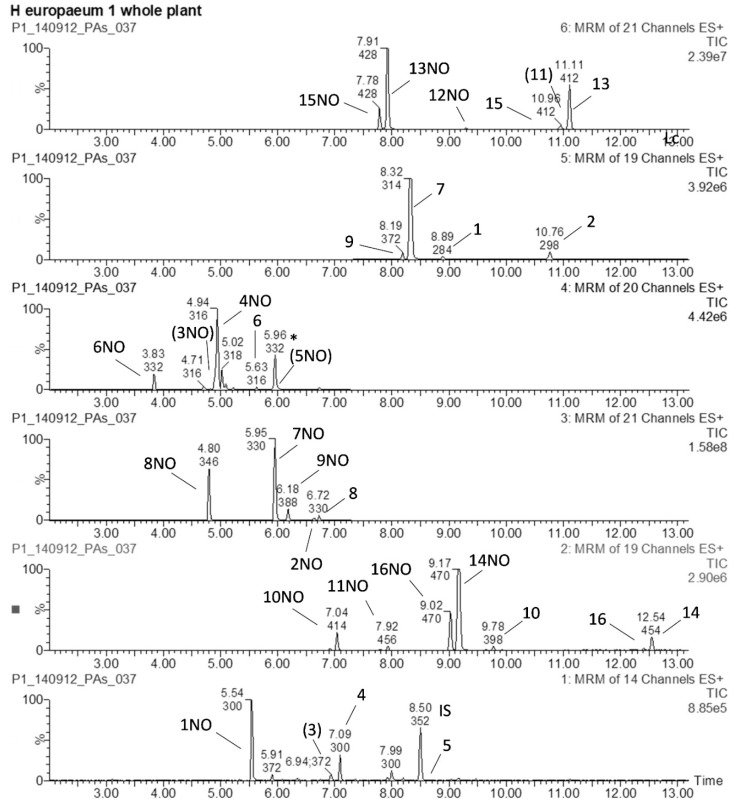
LC-MS/MS multiple reaction monitoring (MRM) chromatograms of a *H. europaeum* extract (whole plant). Annotation is as shown in Figure 1. NO = the N-oxide form. * denotes the ^13^C2-isotopomer of 7NO. IS = internal standard. Compounds between brackets are masked in the chromatogram.

In this study, a total of 31 different PAs (15 free base forms and 16 *N*-oxides) were detected in the three species. Most of these compounds have been described for the three closely related species as discussed above. The chemical structures of the free base PAs present in the three species are given in Figure 1, while their corresponding *N*-oxide forms can be derived by covalently attaching an oxygen atom to the nitrogen atom at position 4. The 7-angeloylheliotrine free base was not detected in the plant extracts. LC-MS/MS chromatograms representative of a *H. europaeum* extract are shown in Figure 2. 

Derivatives of rinderine and heliosupine have so far been reported only in *H. indicum, H. peruvianum, H. transalpinum, H. supinum* and *H. europaeum* [3,17]. The identity of rinderine could be confirmed by comparison with an authentic standard, but a definite assignment of the rinderine derivatives could not be made, since the obtained mass fragmentation spectra could not exclude the possibility of the echinatine structure. However, based on the fact that (+)-trachelanthic acid, which is the necic acid in rinderine, has the same stereochemistry as heliotric acid, the necic acid in heliotrine, its presence was considered more likely than the echinatine structure, which is esterified with (−)-viridifloric acid and for which no heliotrine counterpart is known. Similarly, the definitive structure for the less abundant isomer of lasiocarpine and 5′-acetyllasiocarpine could not be derived, but it is highly plausible that it contains a tigloyl ester at C-7 instead of the angeloyl ester. 

### 2.2. Distribution and Composition of PAs in Heliotropium Species

The flowers of all three species revealed the highest PAs concentration (Table 1). The PAs concentration in the roots of *H. europaeum* was up to 3 times higher than in the corresponding roots of *H. suaveolens* and *H. rotundifolium* (Table 1). No significant differences in the PAs levels were observed between the leaves and stems in the three species. The PA tissue distribution in *H. europaeum* roughly resembles the organ-specific distribution of several *Heliotropium* species (*H. curassavicum*, *H. spathulatum* and *H. indicum*) reported in earlier studies [29,30]. Preferential accumulation of PAs in the flowers is also known from other species, e.g., *Senecio vulgaris* [31]. All major organs of the three *Heliotropium* species revealed similar abundance of free PA bases and PA-*N*-oxides, with PA-*N*-oxides constituting more than 94% of the total PAs in all major tissues analyzed. 

The detected PAs and their corresponding *N*-oxides were classified according to their necine base and the type and number of esterified necic acids as follows: 

The supinine-type PAs included supinine, heleurine and their corresponding *N*-oxides; the heliotrine-type PAs included echinatine, rinderine, 5′-hydroxy-rinderine, 7-acetyl-rinderine, heliotrine, europine, 5′-acetyl-europine and their corresponding *N*-oxides; and the lasiocarpine-type PAs comprised heliosupine, 3′-acetyl-heliosupine, lasiocarpine, iso-lasiocarpine, 5′-acetyl-lasiocarpine, iso-acetyl-lasiocarpine and their corresponding *N*-oxides as well as 7-angeloylheliotrine-*N*-oxide (Figure 1).

**Figure 3:**
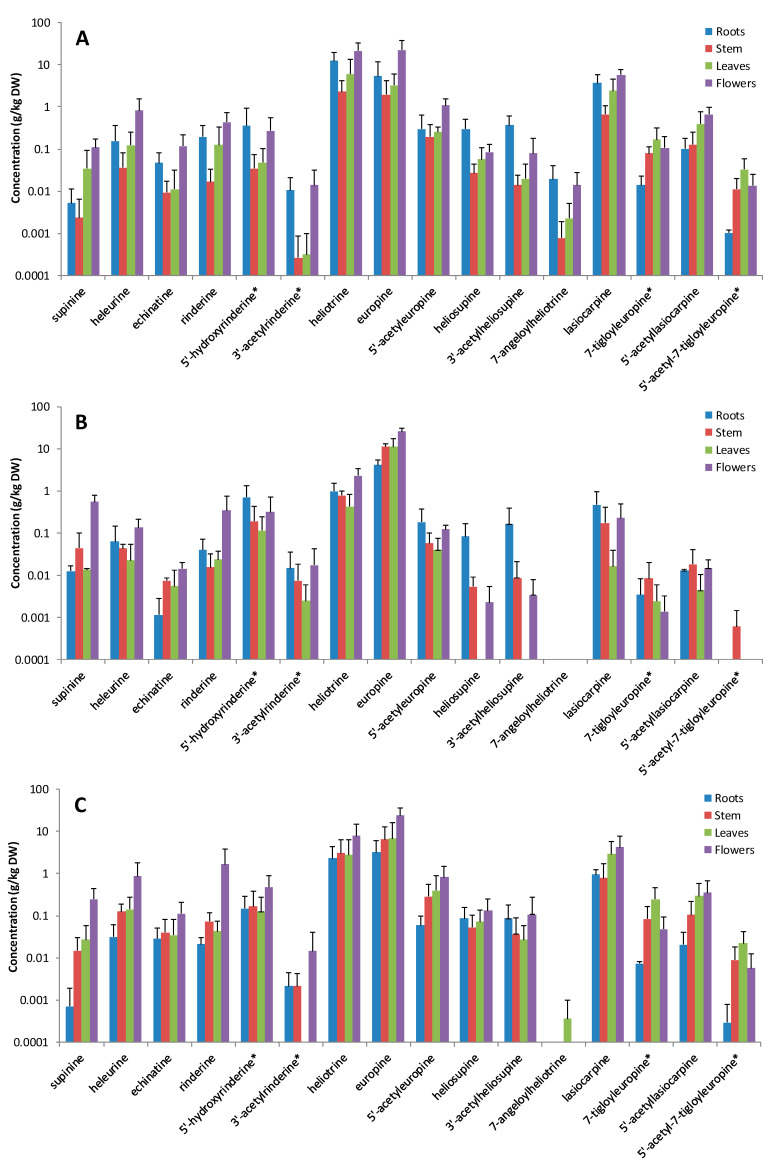
PA composition and the log concentration (µg/g) of the major plant parts (root, stem, leaf and flowers) in (**A**). *H. europaeum* (*n* = 5), (**B**) *H. rotundifolium* (*n* = 5) and (**C**) *H. suaveolens* (*n* = 5). *: tentative identification. Concentrations are for the sum of tertiary amines and corresponding *N*-oxides.

*H. europaeum* and *H. suaveolens* revealed a similar pattern of relative PA abundances with heliotrine-type PAs and lasiocarpine-type Pas, accounting for 72–89% and 8.7–30% of total Pas, respectively (Table 1). *H. rotundifolium*, however, displayed a markedly different PAs profile, with more than 90% of the total PAs found in all major plant tissues belonging to the heliotrine-type PAs. The supinine-type PAs comprised only a small proportion in all tissues analyzed (<4%). The PA compositions in each of the major plant tissues of the three *Heliotropium* species studied are illustrated in Figure 3 and depicted in Supplementary Table S4. The most abundant PAs found in all plant tissues were europine-*N*-oxide, heliotrine-*N*-oxide and lasiocarpine-*N*-oxide, constituting more than 85% of the total PAs in accordance with the study results published by O′Dowd and Edgar [22]. The most prominent PA in all the major plant tissues of *H. rotundifolium* and *H. suaveolens* was europine-*N*-oxide (60–83% for *H. rotundifolium* and 43–57% for *H. suaveolens*) followed by heliotrine-*N*-oxide (3–14% for *H. rotundifolium* and 16–29% for *H. suaveolens*) and lasiocarpine-*N*-oxide (0.1–5% for *H. rotundifolium* and 5–18% for *H. suaveolens*). However, the stems and flowers of *H. europaeum* revealed comparable concentrations of heliotrine-*N*-oxide and europine-*N*-oxide, while the mean relative abundance of heliotrine-*N*-oxide in the roots and leaves was about twice the relative abundance of europine-*N*-oxide (Supplementary Table S4). 

Europine-*N*-oxide was the only alkaloid displaying a statistically significant interaction between major plant tissues and *Heliotropium* species for all tissues analyzed (F = 3.157, Df = 6, *p* < 0.01; Supplementary Table S4). 

### 2.3. Relative Risk Assessment of the Aerial Parts of Heliotropium Species

The toxic potential of PA-producing weeds depends not only on the total PA concentrations in the plant, but also on the relative abundance of the various individual PAs, due to their differences in their toxic potential, as was demonstrated in various studies [9,10]. For instance, 1,2-unsaturated di-esters such as lasiocarpine-type PAs were demonstrated to be substantially more toxic than the 1,2-unsaturated mono-esters, such as heliotrine-type PAs and supinine-type PAs [3,9,10]. 

The lasiocarpine-type PAs content of *H. europaeum* was significantly higher than the corresponding content in *H. rotundifolium* (*p* < 0.05), whereas the remaining multiple comparisons between the three species in relation to their PA content revealed no significant differences (Table 2). Notwithstanding, the total PA content of the aerial parts of the plant among the three species was not significantly different, which could be attributed to the relatively high variability of the PA content within each species (Table 2). Congruent with the lasiocarpine-type content, the relative toxic potency, obtained by the summation of the product PA type content and the iREP, of *H. europaeum* was significantly higher than the corresponding value of *H. rotundifolium*, whereas no statistical differences were observed for the remaining multiple comparisons (Table 2). The heliotrine- and lasiocarpine-type PAs contributed more than 98% to the relative toxic potency of the *Heliotropium* species studied, while the contribution of the supinine-type PAs was negligible (≤1.5%), due to lower abundance and relative toxicity. Although *H. suaveolens* displayed a similar PA content and composition to *H. europaeum*, no statistical difference was found in the relative toxic potency of *H. suaveolens* and *H. rotundifolium* (Table 2). It is reasonable to assume that the relatively low sample size (*n* = 5) limited the chance of detecting a true difference in the total PA content as well as in the relative toxic potency between *H. rotundifolium* and *H. suaveolens*.

## 3. Materials and Methods

### 3.1. Materials, Reagents and Standards

Formic acid (analytical grade) and ammonia solution (25%, analytical grade) were obtained from Merck (Darmstadt, Germany). Acetonitrile (LC-MS grade) was from Biosolve (Valkenswaard, The Netherlands). Analytical PA standards (7-acetylintermedine, echimidine, echinatine, europine, heliosupine, heliotrine, intermedine, lasiocarpine, rinderine, and their corresponding N-oxides) were obtained from Phytoplan (Heidelberg, Germany), except for heliotrine, which was obtained from Latoxan (Valence, France). The purity of the analytical standards was 95% or higher, except for heliotrine that contained approximately 10% europine. A semi-quantitative result was obtained for PAs, lacking a reference standard, by comparison of the peak area with that of a structurally related standard, as indicated in Supplementary Table S1.

### 3.2. Plant Material

Several individual plant samples of *H. europaeum*, *H. rotundifolium* and *H. suaveolens* were collected in August 2014 (*n* = 5) during their flowering stage on a pastoral land in the Galilee region near Kibbutz Gazit, Israel (32°38′15″ N 35°26′49.55″ E). The annual rainfall at the site of collection averaged 577 mm. To account for possible diurnal variations in alkaloid accumulation, plants were harvested between 12:00 and 14:00 p.m. On the same day of collection, the plants were air dried at 50 °C in an oven for 12 h and the major plant organs (inflorescence, leaves, stems and roots) were separately milled to a particle size of <1 mm (Foss Cyclotech 1093, Hillerød, Denmark). The samples were stored in air-tight plastic bags at −20 °C until further processing for LC-MS/MS analysis.

### 3.3. Plant Description

*Heliotropium* species are annual herbs growing from a taproot and reaching maximum heights near 70 cm. Due to their high morphological resemblance, species identification is often only possible by expert botanists. *H. europaeum* is a small to medium-sized annual plant, up to 40 cm high, with a distribution habitat in Israel ranging from the north to the southern Negev [19,20,21]. The stems and leaves are covered with short and dense hairs, equal in length. The flowers are 2 mm in diameter, white with a small yellow spot around the pharynx, and the petals are rounded, egg-shaped. The fruit consists of 2–4 irregularly roughened nutlets. The flowering time of *H. europaeum* is between May and October [19].

*H. suaveolens* is a small to medium-sized weed (height up to 30 cm), distributed in Israel mainly on the coastal plain and on the basalt soil in the Golan Heights. The leaves are egg-shaped and display a thin loose cover of short, equally sized trichrome, bestowing them a delicate silvery hue. The flowers are 3–5 mm in diameter, white and have a small, inconspicuous yellow spot in the pharyngeal tube with rounded petal lobes. In a cross-section, the petiole appears round and hairless on the inside and has sparse hairs on the outside. The flowers give off a delicate and pleasant scent, hence the name of the species. The fruit consists of 2–4 irregularly roughened nutlets. The flowering time of *H. suaveolens* is between May and December [19].

*H. rotundifolium* is a graying shrub with a solid base, distributed in Israel throughout the country mostly in limestone hills and rock outcrops, but is mostly absent in the southern Negev. The leaves are broad-egg-shaped and round. The stems and leaves are covered with short, dense hairs that impart the whole plant with a strong silvery-gray hue. The inflorescences are long and multi-flowered. The flowers are of 4 mm in diameter and white, having a small yellow spot at the opening of the flower tube. The petioles are triangular-pointed, with the tip bent and facing the center of the flower. In addition, the petiole is hairy on the outside and completely hairless on the inside. The flowering time of *H. rotundifolium* is between April and September [19].

### 3.4. LC-MS/MS Analysis for PA and PA-N-Oxides in Heliotropium Species

Dried, ground plant material was extracted using a slightly adapted procedure described by Shimshoni et al. [17]. Jacobine was added as an internal standard to check the sample extraction procedure and the analytical performance of LC-MS/MS. Powdered material was extracted with 2% formic acid solution in a 1 to 100 ratio (*w/v*). The extract was then filtered and 5 µL was diluted 200 times with 2 mM NH_4_OH solution in autosampler vials. PA composition and content of the samples were determined using a Waters Acquity chromatographic system coupled to a Waters Quattro Premier XE tandem mass spectrometer (Waters, Milford, MA, USA), run in multiple reaction monitoring mode (MRM) with positive electrospray ionization. Cone voltage was set at 30 V, desolvation gas temperature at 450 °C, source block temperature at 120 °C and the argon collision gas pressure at 4.0 × 10^−3^ mbar. Dwell time of the selected transitions was 10 ms with an interscan delay of 0.5 ms. Separation of PAs was accomplished on a Waters UPLC BEH C18 (1.7 µm, 150 × 2.1 mm) analytical column (Waters, Milford, MA, USA), kept at 50 °C and run at 0.4 mL/min. A mobile phase consisting of 6.5 mM NH_4_OH in water and 6.5 mM NH_4_OH in acetonitrile was used. The gradient started at 100% water and was changed linearly to 50% acetonitrile in 12 min. After 0.2 min, the composition was returned to the starting condition and the column was allowed to equilibrate for another 2.5 min. Mass spectrometric data were processed using Masslynx software, version 4.1 (Waters, Milford, MA, USA).

Representative samples of each species were screened for the presence of novel PAs using parent ion scanning. Typical product fragments were selected such as 120, 122, 138, 156, 172 and 254 *m/z*. Mass scanning range was from 200 to 500 *m/z* with a mass resolution of 0.1 D and a scan time of 800 ms. Spectra were recorded using a fixed cone voltage of 30 V and fixed collision energy of 30 eV. Compounds producing a protonated molecular ion with an even mass and displaying fragmentation behavior typical for (specific types of) PAs were tentatively identified as PAs. These potential PAs were further identified by collecting individual fragmentation spectra at a collision energy range (20–40 eV). Based on the fragmentation spectra and comparison with literature data [3,7], it was possible to assign chemical structures to the majority of PAs present in the extracts. As isomeric PAs (e.g., rinderine and echinatine) often produce identical fragmentation spectra, it was not possible to discriminate between isomers and, consequently, those assignments are tentative. Supplementary Material S3 presents representative mass spectra of the identified PAs in the *Heliotropium* extracts, including a tentative assignment of the major fragments present in the mass spectra. All (tentatively) identified compounds have been included in the MRM method, by selecting two product ions (typically the most abundant ones). See Supplementary Table S1 for the mass spectrometric settings selected for each compound. PAs were quantified against a range of calibrant samples (0–500 ng/mL) containing the available PA standards. Limit of quantification in the plant materials was 1 µg/g.

### 3.5. Comparative Risk Assessment 

The comparative risk assessment was performed only for the aerial parts of the plant (sum of stem, leaves and inflorescence), since only aerial parts are usually co-harvested with edible crops. The concentration of each congener of the aerial parts of each *Heliotropium* species was multiplied by its corresponding iREP factor [9]. Summing up these products for each species yielded the overall sum of equivalents per species, e.g., relative toxic potency (Table 2). The sum was then considered as a suitable parameter quantitatively describing the relative toxic potential of each species. According to Merz and Schrenk, the following iREP criteria for the various classes of PAs were used: 7*S*-mono-esters 0.3 (rinderine- and heliotrine-type) and open-chained 7*S*-di-esters 1.0 (heliosupine- and lasiocarpine-type) [9]. The corresponding *N*-oxides were assigned the same iREP values as the corresponding PAs, due to their rapid conversion to their corresponding free base under physiological conditions [32,33].

### 3.6. Statistical Analysis

Mean values for each PA were determined from 5 replicates of each major organ plant of the three species (flowers, leaves, stems and roots). Differences in PA concentrations among the three *Heliotropium* species were tested for the same major plant organ (interaction of species with major plant organ) by using a two-way ANOVA test, followed by a Bonferroni multiple comparison test. A *p* value of < 0.05 was considered to be significant. Differences in the relative toxic potential (sum of the products of concentration and iREP factor) and the content of PA types between the three *Heliotropium* species were determined using one-way ANOVA, followed by the Tukey multiple comparison test. A *p* value of < 0.05 was considered to be significant.

The statistical tests were performed with GraphPad Prism version 5.04 for Windows (GraphPad Software, La Jolla, CA, USA).

## 4. Conclusions

In this study, LC-MS/MS was used to analyze PAs in the major organ parts of *H. europaeum, H. rotundifolium* and *H. suaveolens.* The application of LC-MS/MS for the evaluation of PA profiles is expected to further expand the PA composition of known PA-producing plants. Based on the interim relative potency factor concept, *H. europaeum* was found to pose a higher health risk to exposed animals and humans as compared to *H. rotundifolium*, whereas no differentiation in the relative toxic potency could be established for *H. suaveolens* and *H. rotundifolium* as well as for *H. suaveolens* and *H. europaeum*. 

## Figures and Tables

**Table 1:** PA content of the major plant parts of *H. europaeum*, *H. rotundifolium* and *H. suaveolens*. The PAs were classified according to the PA necine base and the number and type of esterifying necic acids.

	*H. europaeum* ^a^				*H. rotundifolium* ^b^				*H. suaveolens* ^c^			
	*Root*	*Stem*	*Leaf*	*Flower*	*Root*	*Stem*	*Leaf*	*Flower*	*Root*	*Stem*	*Leaf*	*Flower*
Total PA content (mean ± SD) (g/kg dry weight)	23 ± 13 *	5 ± 5	13 ± 12	53 ± 15^**^	7 ± 3	12 ± 2	11 ± 7	29 ± 7 **	7 ± 5	11 ± 10	13 ± 17	40 ± 20 **
% Total PA bases	5.8 ± 3.2	4.2 ± 1.7	3.4 ± 2.1	4.2 ± 1.5	2.9 ± 0.1	2.7 ± 2.0	1.5 ± 0.2	4.2 ± 0.7	5.7 ± 3.1	2.2 ± 1.0	2.5 ± 0.3	4.2 ± 1.9
% Total PA-*N*-oxides	94.2 ± 3.2	95.8 ± 1.7	96.6 ± 2.1	95.8 ± 1.5	97.1 ± 0.1	97.3 ± 2.0	98.5 ± 0.2	95.8 ± 0.7	94.3 ± 3.1	97.8 ± 1.0	97.5 ± 0.3	95.8 ± 1.9
% Supinine-type	0.5 ± 0.4	0.7 ± 0.3	1.2 ± 0.5	1.7 ± 0.8	0.9 ± 0.8	0.7 ± 0.5	0.3 ± 0.1	2.2 ± 0.5	0.5 ± 0.6	1.7 ± 0.9	3.4 ± 4.2	2.3 ± 1.3
% Heliotrine-type	76 ± 11	82 ± 6	75 ± 14	86 ± 7	90.3 ± 8.3 ^d^	97.7 ± 1.5 ^d^	99.6 ± 0.3 ^d^	97.0 ± 1.3 ^d^	79 ± 11	89 ± 7	73 ± 22	85 ± 15
% Lasiocarpine-type	23 ± 12	17 ± 6	24 ± 14	12 ± 7	8.8 ± 7.5 ^e^	1.5 ± 2.0 ^e^	0.1 ± 0.2 ^e^	0.7 ± 0.8	21 ± 9	9 ± 7	24 ± 25	13 ± 14

^a^ PA levels in flowers are significantly higher as compared to the other plant parts within *H. europaeum* (** *p* < 0.01). PA levels in roots are higher than in the stem (* *p* < 0.05). ^b^ PA levels in flowers are significantly higher as compared to the other plant parts within *H. rotundifolium* (** *p* < 0.01). ^c^ PA levels in flowers are significantly higher as compared to the other plant parts within *H. suaveolens* (** *p* < 0.01). ^d^ Relative abundances of heliotrine-type PAs in the root, stem and leaf of *H. rotundifolium* are significantly higher as compared to the relative abundances of heliotrine-type PAs in the corresponding plant parts of *H. europaeum* (*p* < 0.05, t = 2.97; *p* < 0.01, t = 3.18; *p* < 0.001, t = 5.05, respectively). Relative abundances of heliotrine-type PAs in the leaf and flowers of *H. rotundifolium* are significantly higher as compared to the relative abundances of heliotrine-type PAs in the corresponding plant parts of *H. suaveolens* (*p* < 0.001, t = 5.59; *p* < 0.05, t = 2.56, respectively). ^e^ Relative abundances of lasiocarpine-type PAs in the root, stem and leaf of *H. rotundifolium* are significantly lower as compared to the relative abundances of lasiocarpine-type PAs in the corresponding plant parts of *H. europaeum* (*p* < 0.05, t = 2.89; *p* < 0.05, t = 3.05; *p* < 0.001, t = 4.63, respectively). Relative abundance of lasiocarpine PAs in the leaf of *H. rotundifolium* is significantly lower as compared to the relative abundance of PAs in the corresponding plant part of *H. suaveolens* (*p* < 0.001, t = 4.71).

**Table 2:** Relative toxic potencies of *H. europaeum, rotundifolium* and *suaveolens*, based on their average PA content in the aerial part of the plant (leaves, stems and flowers) and the corresponding relative toxic equivalent of each PA type.

	PA Content of Aerial Part ^a^ in g/kg			Interim Relative Potency Factor (iREP)	Relative Toxic Potency (Concentration x iREP)		
	*H. europaeum*	*H. rotundifolium*	*H. suaveolens*		*H. europaeum*	*H. rotundifolium*	*H. suaveolens*
Supinine-type content	0.4 ± 0.3 (1.4%)	0.3 ± 0.5 (1.7%)	0.5 ± 0.8 (2%)	0.3	0.12 ± 0.09 (1%)	0.09 ± 0.15 (1.5%)	0.15 ± 0.2 (1.5%)
Heliotrine-type content	24.5 ± 15 (83.3%)	16.6 ± 7.2 (97.1%)	20.8 ± 12 (85%)	0.3	7.3 ± 4.1 (62%)	5.0 ± 2.1 (95.2%)	6.2 ± 4.4 (65%)
Lasiocarpine-type content	4.5 ± 2.1 (15.3%)	0.2 ± 0.1 * (1.1%)	3.2 ± 2.2 (13%)	1	4.5 ± 2.2 (37%)	0.2 ± 0.1 (3.3%)	3.2 ± 2.3 (33.5%)
Sum of PA/relative toxic potency ^b^	29.4 ± 15	17.1 ± 7.1	24.5 ± 12	-	11.9 ± 4.4	5.3 ** ± 2.2	9.5 ± 5.1

^a^ The relative toxic potency was determined only for the aerial part of the plants (leaves, stems and flowers), since only aerial parts were found in crops intended for animal consumption such as hay. Percentage of each PA type is provided in parenthesis. ^b^ Sum of PA content included the PA bases as well as their corresponding *N*-oxides. * The lasiocarpine-type PA content of *H. europaeum* was significantly different from the lasiocarpine-type PA content of *H. rotundifolium* (*p* < 0.01). No statistical differences were observed for the remaining comparison groups. ** The relative toxic potency of *H. rotundifolium* was significantly lower than *H. europaeum* (*p* < 0.04). No statistical differences were observed between the relative toxic potencies of *H. europaeum* and *H. suaveolens* as well as between *H. suaveolens* and *H. rotundifolium*.

## Data Availability

Data is contained within the article or supplementary material.

## Supplementary Materials

The following are available online: Table S1: Mass spectrometric conditions used and indicative retention times of standards used for pyrrolizidine alkaloids detected in *Heliotropium* species, Table S2: Pyrrolizidine alkaloids identified in this study in *Heliotropium* species., Data file S3: MS/MS fragmentation spectra of pyrrolizidine alkaloids detected in *Heliotropium* species. Collision energy applied is 25 eV, Table S4: Relative PA abundance (%) present in the major plant parts of *H. europaeum*, *H. rotundifolium* and *H. suaveolens*.

## Sample Availability

Samples of the compounds are not available from the authors.
