# Supplementary material for: Comparative Risk Assessment of Three Native Heliotropium Species in Israel
Source: Molecules. 2021 Jan 28;26(3):689. doi: 10.3390/molecules26030689 (PMC7866218; doi:10.3390/molecules26030689)
Supplement: Supplementary file 1 [file molecules-26-00689-s001.pdf]

Supplementary files

Comparative Risk Assessment of Three Native *Heliotropium* Species in Israel

Jakob A. Shimshoni [1,*], Shimon Barel [2] and Patrick P.J. Mulder [3]

[1] Department of Food Quality & Safety, Institute for Postharvest and Food Sciences, Agricultural Research Organization, Volcani Center, Rishon LeTsyion 7528809, Israel
[2] Department of Toxicology, Kimron Veterinary Institute, Bet Dagan 50250, Israel; Shimonba@moag.gov.il
[3] Wageningen Food Safety Research, Wageningen University & Research, PO Box 230, 6700 AE Wageningen, The Netherlands; patrick.mulder@wur.nl
* Correspondence: jakobs@volcani.agri.gov.il; Tel: +972-50-6243961; Fax: +972-3-9681730

Table S1. Mass spectrometric conditions used and indicative retention times of standards used for pyrrolizidine alkaloids detected in *Heliotropium spp.*

Table S2. Pyrrolizidine alkaloids identified in this study in *Heliotropium spp.*

Data file S3: MS/MS fragmentation spectra of pyrrolizidine alkaloids detected in *Heliotropium ssp*. Collision energy applied is 25 eV.

Table S4. Relative PA abundance (%) present in the major plant parts of *H. Europaeum*, *H. rotundifolium* and *H. suaveolens*.

Table 1. Mass spectrometric conditions used and indicative retention times of standards used for pyrrolizidine alkaloids detected in *Heliotropium spp.*

Pyrrolizidine Alkaloid	Code	Precursor ion (*m*/*z*)	Fragment 1 (*m*/*z*)	Collision energy (eV)	Fragment 2 (*m*/*z*)	Collision energy (eV)	Retention time (min)	Standard used for quantification
Jacobine	IS	352.2	155	30			8.50	
Supinine	1	284.2	122	20	140	25	8.90	Intermedine
Supinine-*N*-oxide	1NO	300.2	120	30	156	30	5.55	Intermedine-*N*-oxide
Heleurine	2	298.2	122	30	140	25	10.75	Heliotrine
Heleurine-*N*-oxide	2NO	314.2	120	30	156	25	6.65	Heliotrine-*N*-oxide
Intermedine[1]	17	300.2	94	35	156	30	6.35	Intermedine
Intermedine-*N*-oxide[2]	17NO	316.2	94	40	172	30	4.75	Intermedine-*N*-oxide
Echinatine	3	300.2	138	30	156	30	6.95	Echinatine
Echinatine-*N*-oxide	3NO	316.2	111	40	172	30	4.90	Echinatine-*N*-oxide
Rinderine	4	300.2	138	30	156	30	7.05	Rinderine
Rinderine-*N*-oxide	4NO	316.2	111	40	172	30	4.95	Rinderine-*N*-oxide
Heliotrine	7	314.2	138	25	156	25	8.30	Heliotrine
Heliotrine-*N*-oxide	7NO	330.2	111	35	172	25	5.95	Heliotrine-*N*-oxide
5'-Hydroxyrinderine*	6	316.2	94	40	138	30	5.65	Rinderine
5'-Hydroxyrinderine-*N*-oxide*	6NO	332.2	111	35	172	30	3.85	Rinderine-*N*-oxide
Europine	8	330.2	94	35	138	30	6.70	Europine
Europine-*N*-oxide	8NO	346.2	172	30	256	25	4.80	Europine-*N*-oxide
7-Acetylintermedine	18	342.2	120	30	138	20	8.65	7-Acetylintermedine
7-Acetylintermedine-*N*-oxide	18NO	358.2	180	30	214	30	6.25	7-Acetylintermedine-*N*-oxide
3'-Acetylrinderine*	5	342.2	120	30	138	20	8.15	7-Acetylintermedine
3'-Acetylrinderine-*N*-oxide*	5NO	358.2	172	30	298	25	5.90	7-Acetylintermedine-*N*-oxide
5'-Acetyleuropine	9	372.2	156	25	254	25	8.20	Europine
5'-Acetyleuropine-*N*-oxide	9NO	388.2	172	30	328	20	6.20	Europine-*N*-oxide
7-Angeloylheliotrine-*N*-oxide	12NO	412.2	136	30	330	25	9.30	Lasiocarpine-*N*-oxide
Echimidine	19	398.2	120	25	220	20	9.90	Echimidine
Echimidine-*N*-oxide	19NO	414.2	254	30	352	25	7.25	Echimidine-*N*-oxide
Heliosupine	10	398.2	120	25	220	20	9.75	Heliosupine
Heliosupine-*N*-oxide	10NO	414.2	94	30	254	30	7.10	Heliosupine-*N*-oxide
Lasiocarpine	13	412.2	120	25	220	20	11.10	Lasiocarpine
Lasiocarpine-*N*-oxide	13NO	428.2	138	30	254	25	7.90	Lasiocarpine-*N*-oxide
7-Tigloyleuropine*	15	412.2	120	25	220	20	10.95	Lasiocarpine
7-Tigloyleuropine-*N*-oxide*	15NO	428.2	138	30	254	25	7.75	Lasiocarpine-*N*-oxide
3'-Acetylheliosupine	11	440.2	120	25	220	20	11.00	Heliosupine
3'-Acetylheliosupine-*N*-oxide	11NO	456.2	254	30	338	25	7.95	Heliosupine-*N*-oxide
5'-Acetyllasiocarpine	14	454.2	120	30	336	25	12.55	Lasiocarpine
5'-Acetyllasiocarpine-*N*-oxide	14NO	470.2	352	20	410	20	9.20	Lasiocarpine-*N*-oxide
5'-Acetyl-7-tigloyleuropine*	16	454.2	120	30	336	25	12.40	Lasiocarpine
5'-Acetyl-7-tigloyleuropine-*N*-oxide*	16NO	470.2	352	20	410	20	9.00	Lasiocarpine-*N*-oxide

[1] Coelutes with its isomer lycopsamine.

[2] Coelutes with its isomer lycopsamine-*N*-oxide.

* Tentative identification, based on fragmentation spectra, MW, RT and comparison with structurally related analytical standards.

Table 2. Pyrrolizidine alkaloids identified in this study in *Heliotropium spp.*

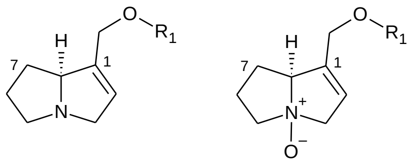

**Pyrrolizidine alkaloid**	***t*-amine**		***N*-oxide**		R1
	Elem. comp.	MW	Elem. comp.	MW	
Supinine (**1**)	$C_{15}H_{25}NO_4$	283.18	$C_{15}H_{25}NO_5$	299.17	(+)-Trachelanthic acid
Heleurine (**2**)	$C_{16}H_{27}NO_4$	297.19	$C_{16}H_{27}NO_5$	313.19	Heliotric acid

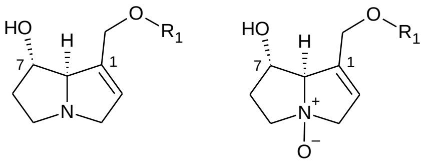

**Pyrrolizidine alkaloid**	***t*-amine**		***N*-oxide**		**R1**
	Elem. comp.	MW	Elem. comp.	MW	
Echinatine (**3**)	$C_{15}H_{25}NO_5$	299.17	$C_{15}H_{25}NO_6$	315.17	(−)-Viridifloric acid
Rinderine (**4**)	$C_{15}H_{25}NO_5$	299.17	$C_{15}H_{25}NO_6$	315.17	(+)-Trachelanthic acid
Heliotrine (**7**)	$C_{16}H_{27}NO_5$	313.19	$C_{16}H_{27}NO_6$	329.18	Heliotric acid
5'-Hydroxyrinderine (**6**)	$C_{15}H_{25}NO_6$	315.17	$C_{15}H_{25}NO_7$	331.16	Echimidinic acid
Europine (**8**)	$C_{16}H_{27}NO_6$	329.18	$C_{16}H_{27}NO_7$	345.18	Lasiocarpic acid
3'-Acetylrinderine (**5**)	$C_{16}H_{27}NO_6$	341.20	$C_{16}H_{27}NO_7$	359.19	(+)-Trachelanthic acid
5'-Acetyleuropine (**9**)	$C_{17}H_{29}NO_7$	371.19	$C_{17}H_{29}NO_8$	387.19	5'-Acetyllasiocarpic acid

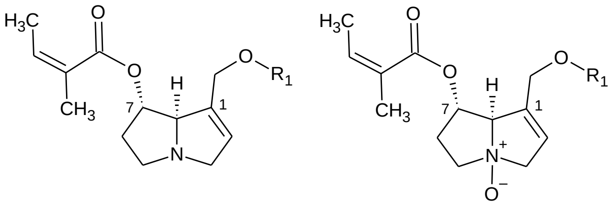

**Pyrrolizidine alkaloid**	***t*-amine**		***N*-oxide**		**R1**
	Elem. comp.	MW	Elem. comp.	MW	
7-Angeloylheliotrine (**12**)	$C_{21}H_{33}NO_6$	395.23	$C_{21}H_{33}NO_7$	411.23	Heliotric acid
Heliosupine (**10**)	$C_{20}H_{31}NO_7$	397.22	$C_{20}H_{31}NO_8$	413.22	Echimidinic acid
Lasiocarpine (**13**)	$C_{21}H_{33}NO_7$	411.23	$C_{21}H_{33}NO_8$	427.22	Lasiocarpic acid
3'-Acetylheliosupine (**11**)	$C_{22}H_{33}NO_8$	439.23	$C_{22}H_{33}NO_9$	455.23	3'-Acetylechimidinic acid
5'-Acetyllasiocarpine (**14**)	$C_{23}H_{35}NO_8$	453.24	$C_{23}H_{35}NO_9$	469.23	5'-Acetyllasiocarpic acid

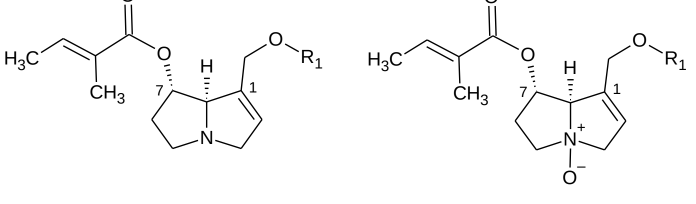

**Pyrrolizidine alkaloid**	***t*-amine**		***N*-oxide**		**R1**
	Elem. comp.	MW	Elem. comp.	MW	
7-Tigloyleuropine (**15**)	$C_{21}H_{33}NO_7$	411.23	$C_{21}H_{33}NO_8$	427.22	Lasiocarpic acid
5'-Acetyl-7-tigloyleuropine (**16**)	$C_{23}H_{35}NO_8$	453.24	$C_{23}H_{35}NO_9$	469.23	5'-Acetyllasiocarpic acid

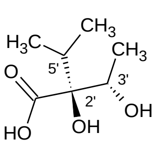

(-)-viridifloric acid

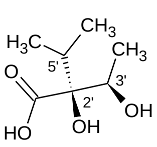

(+)-trachelantic acid

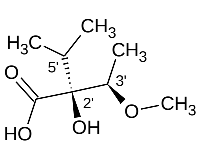

heliotric acid

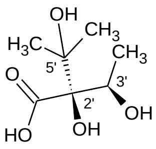

echimidinic acid

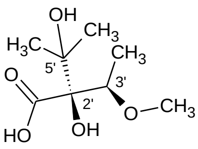

lasiocarpic acid

Data file S3: MS/MS fragmentation spectra of pyrrolizidine alkaloids detected in *Heliotropium ssp*. Collision energy applied is 25 eV.

MS/MS fragmentation spectrum of supinine (1), RT = 8.90 min

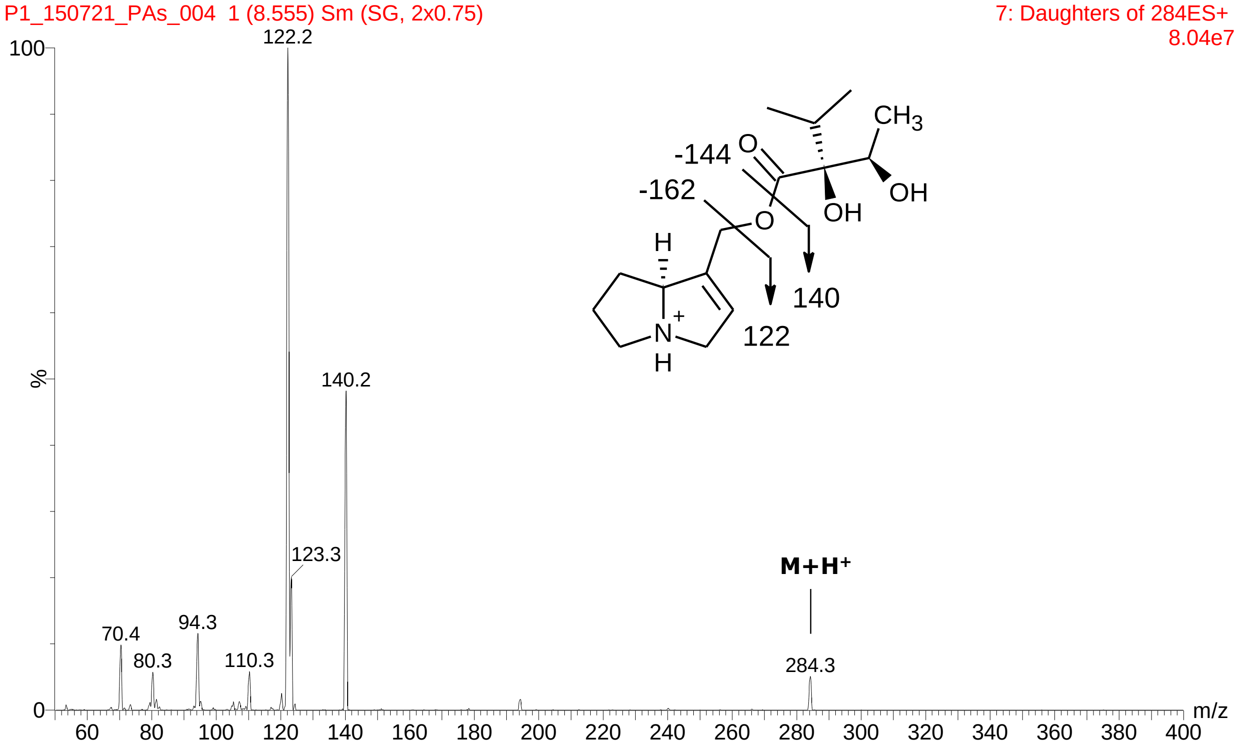

MS/MS fragmentation spectrum of supinine-N-oxide (1NO), RT = 5.55 min

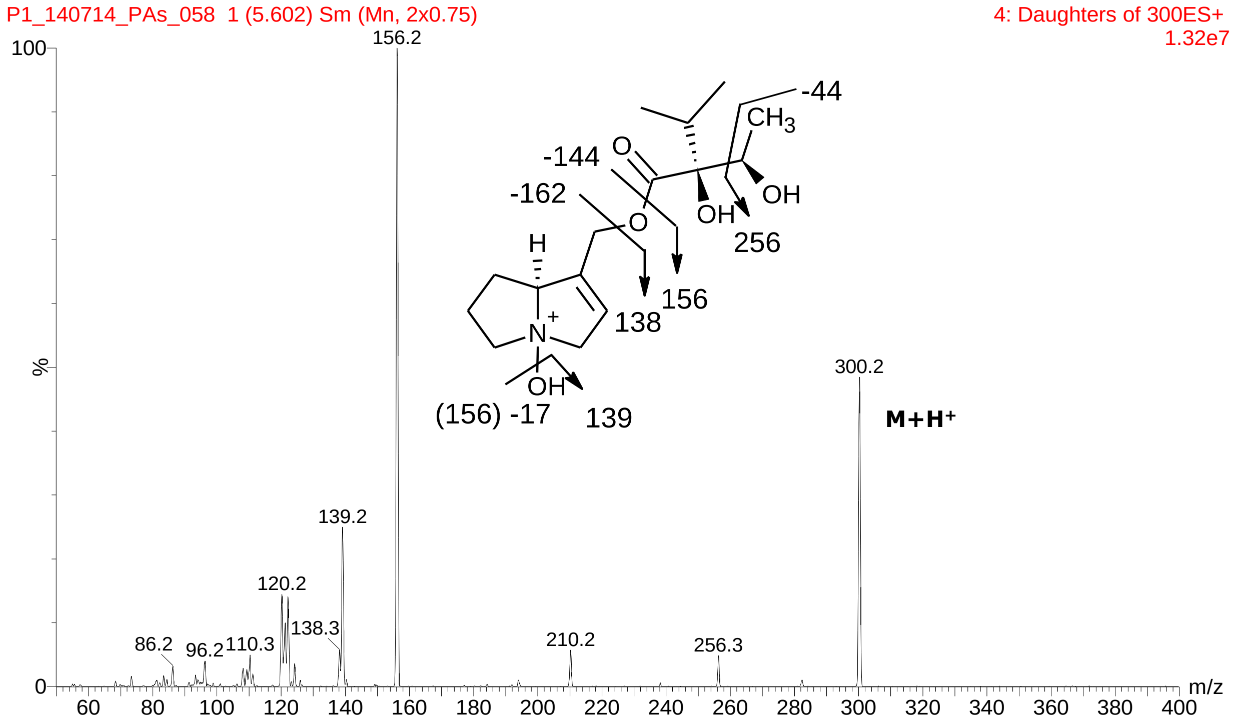

MS/MS fragmentation spectrum of heleurine (2), RT = 10.75 min

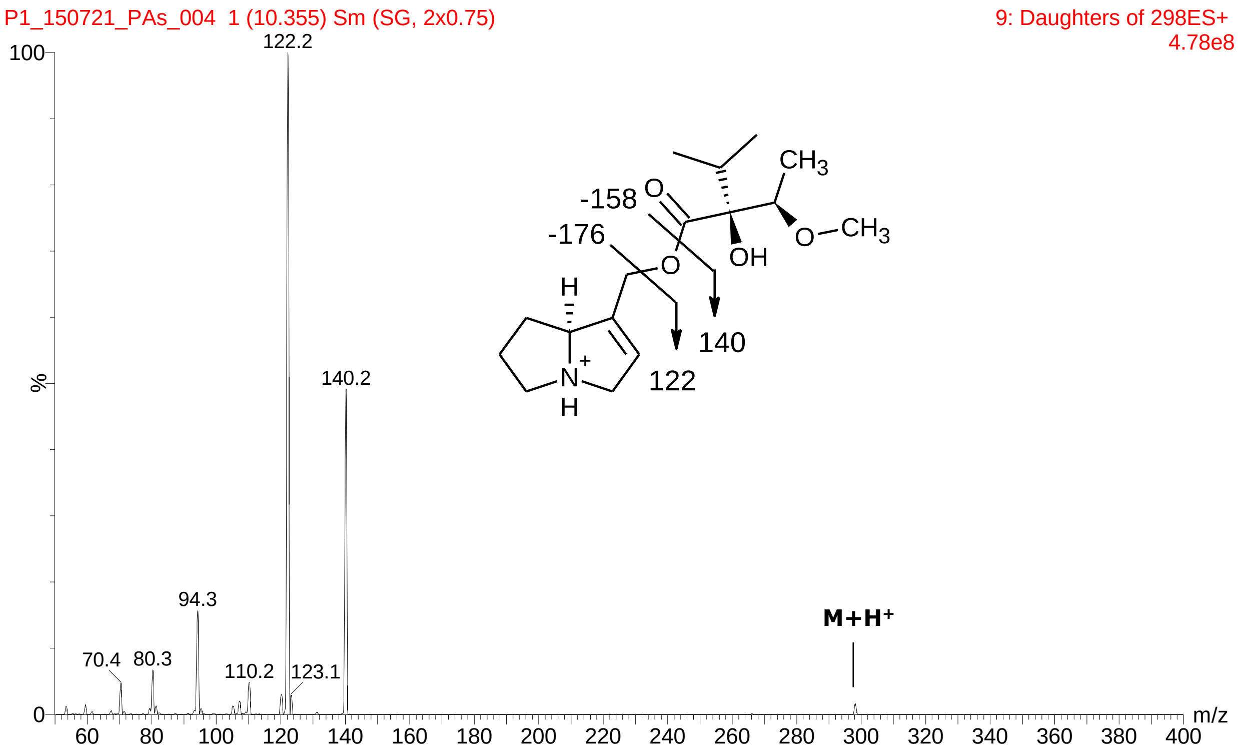

MS/MS fragmentation spectrum of heleurine-N-oxide (2NO), RT = 6.65 min

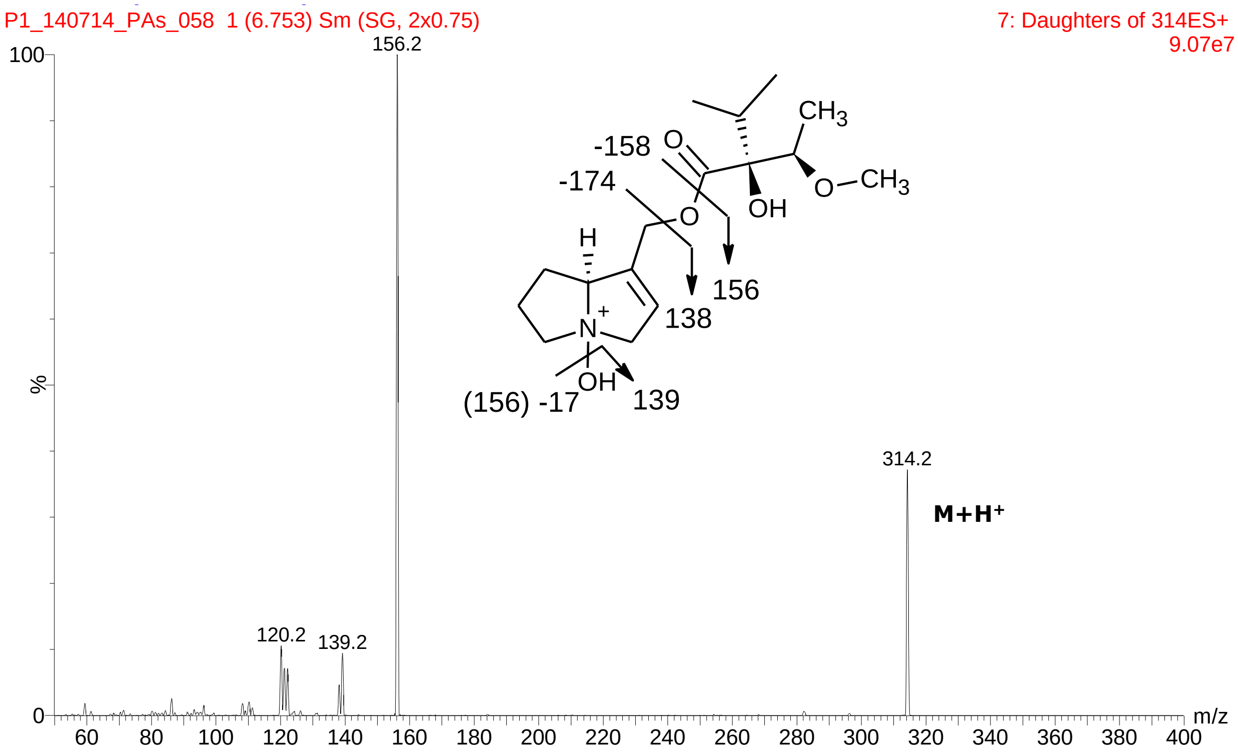

MS/MS fragmentation spectrum of echinatine (3), RT = 6.95 min

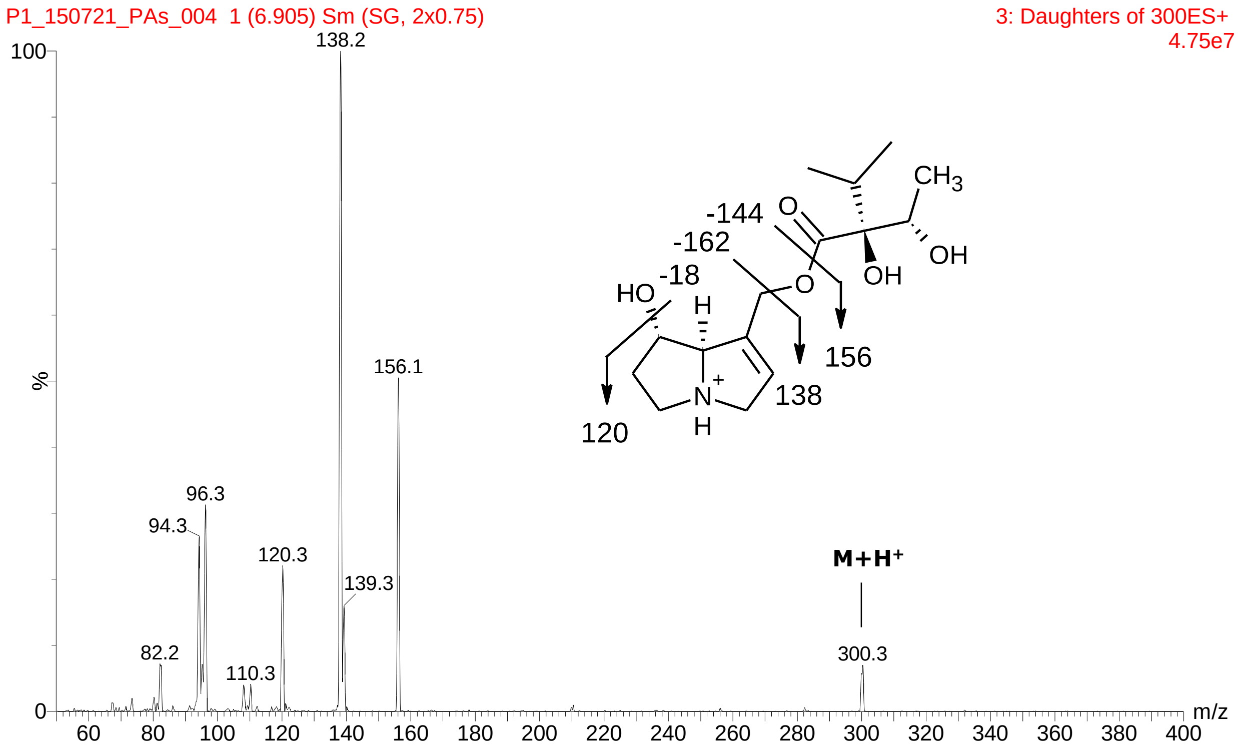

MS/MS fragmentation spectrum of rinderine (4), RT = 7.05 min

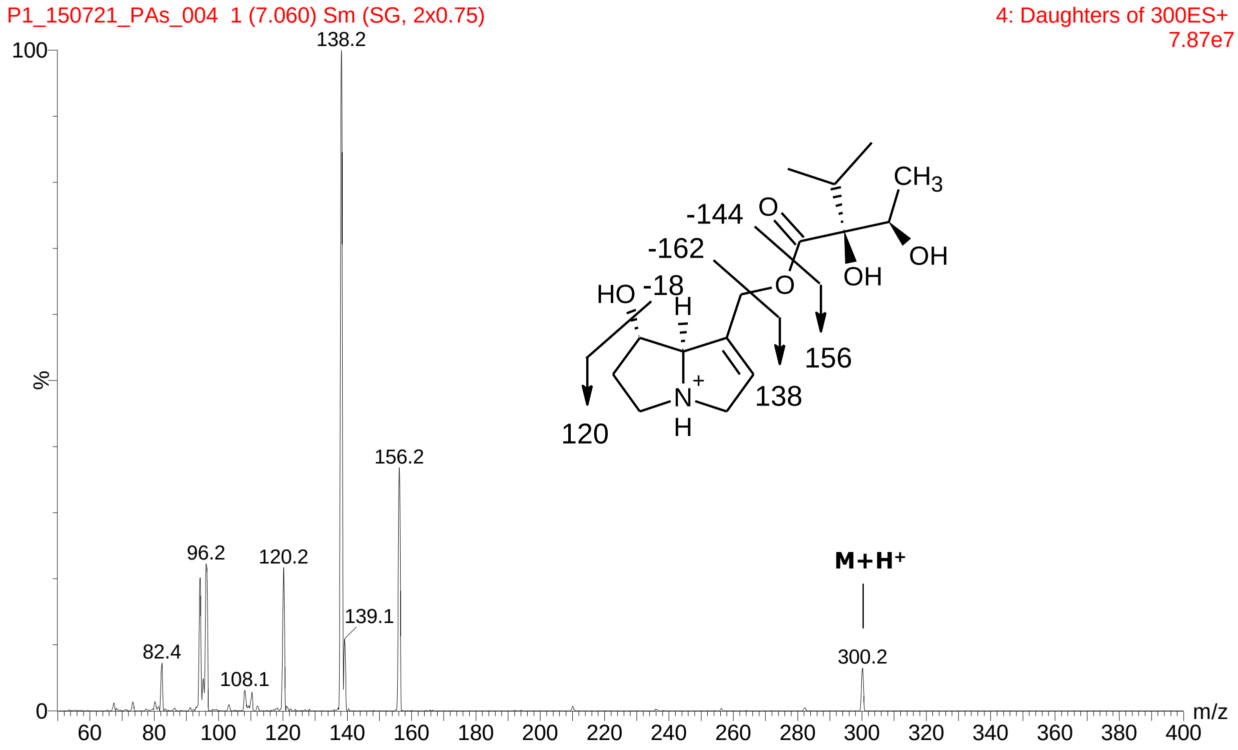

MS/MS fragmentation spectrum of rinderine-N-oxide (4NO), RT = 4.95 min

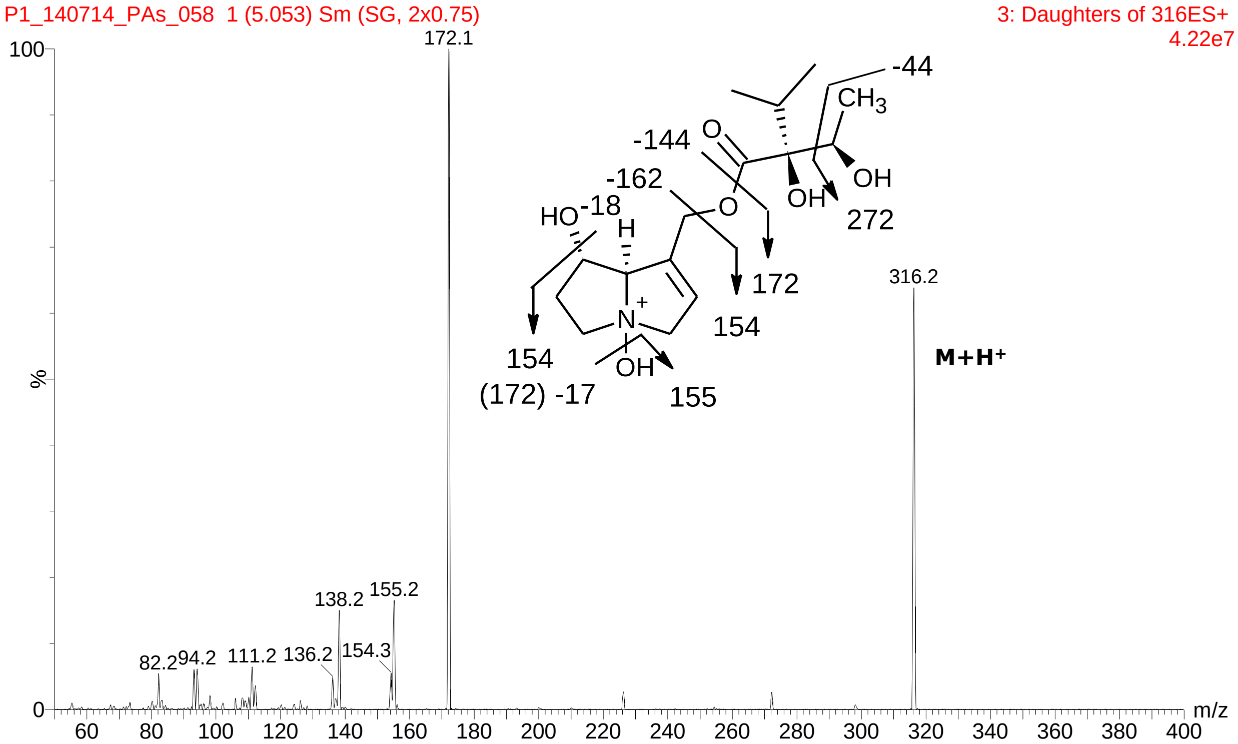

MS/MS fragmentation spectrum of 5'-hydroxyrinderine (6), RT = 5.65 min

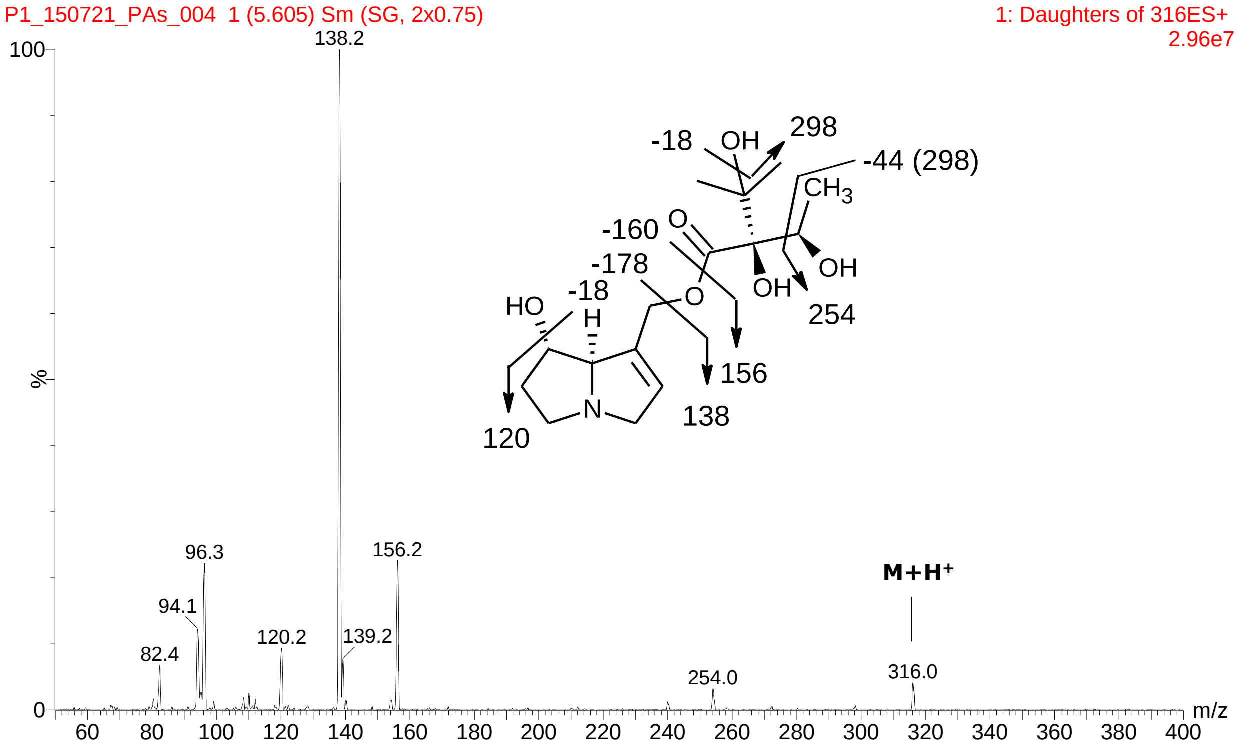

MS/MS fragmentation spectrum of 5’-hydroxyrinderine-N-oxide (6NO), RT = 3.85 min

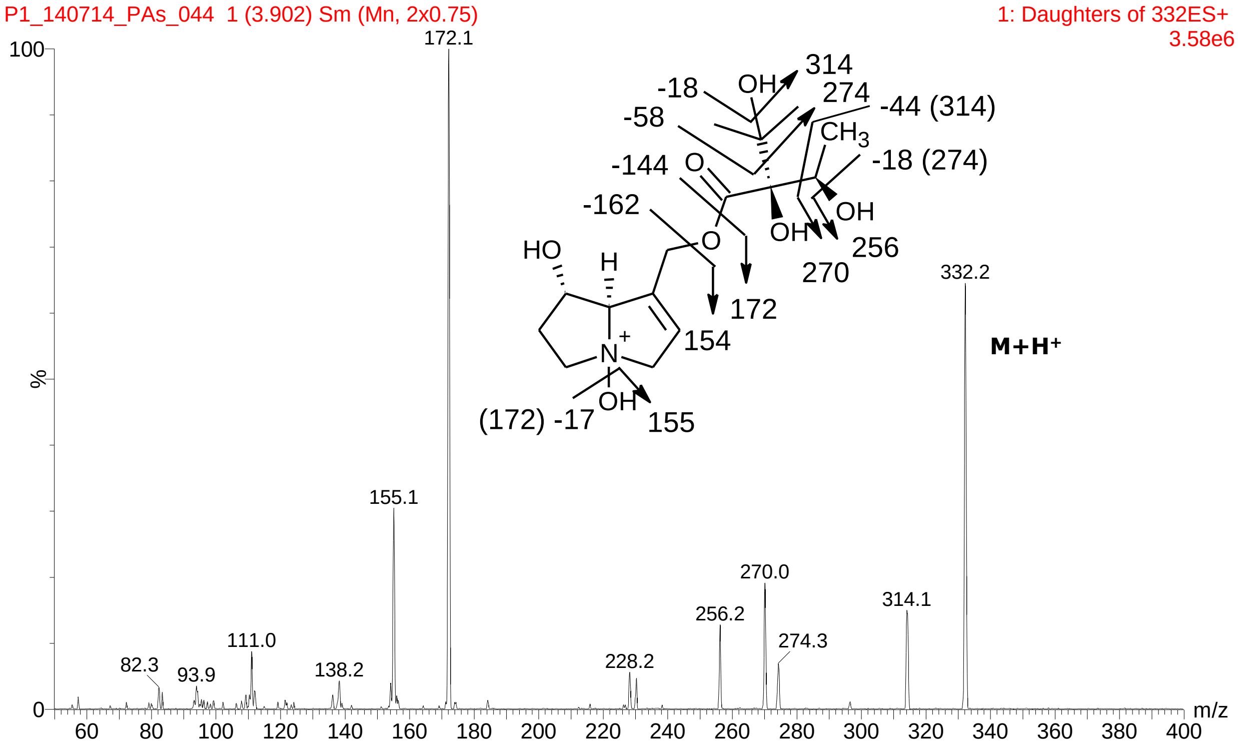

MS/MS fragmentation spectrum of heliotrine (7), RT = 8.30 min

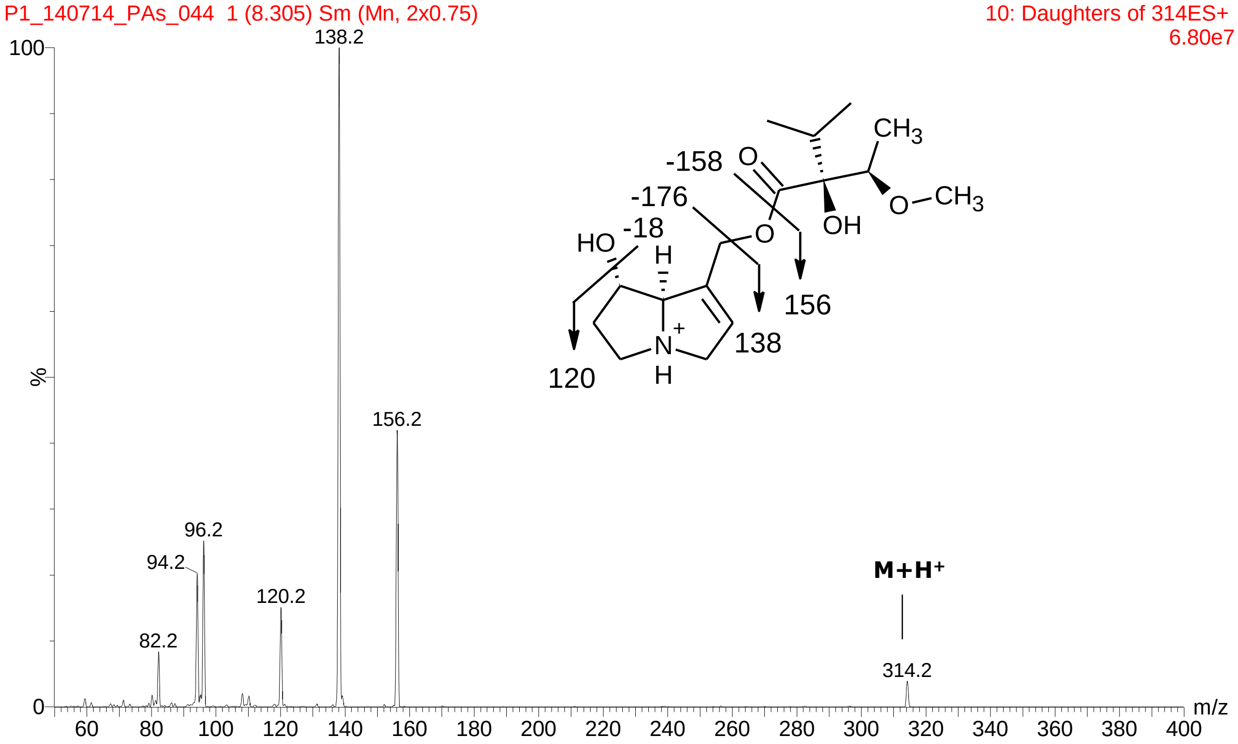

MS/MS fragmentation spectrum of heliotrine-N-oxide (7NO), RT = 5.95 min

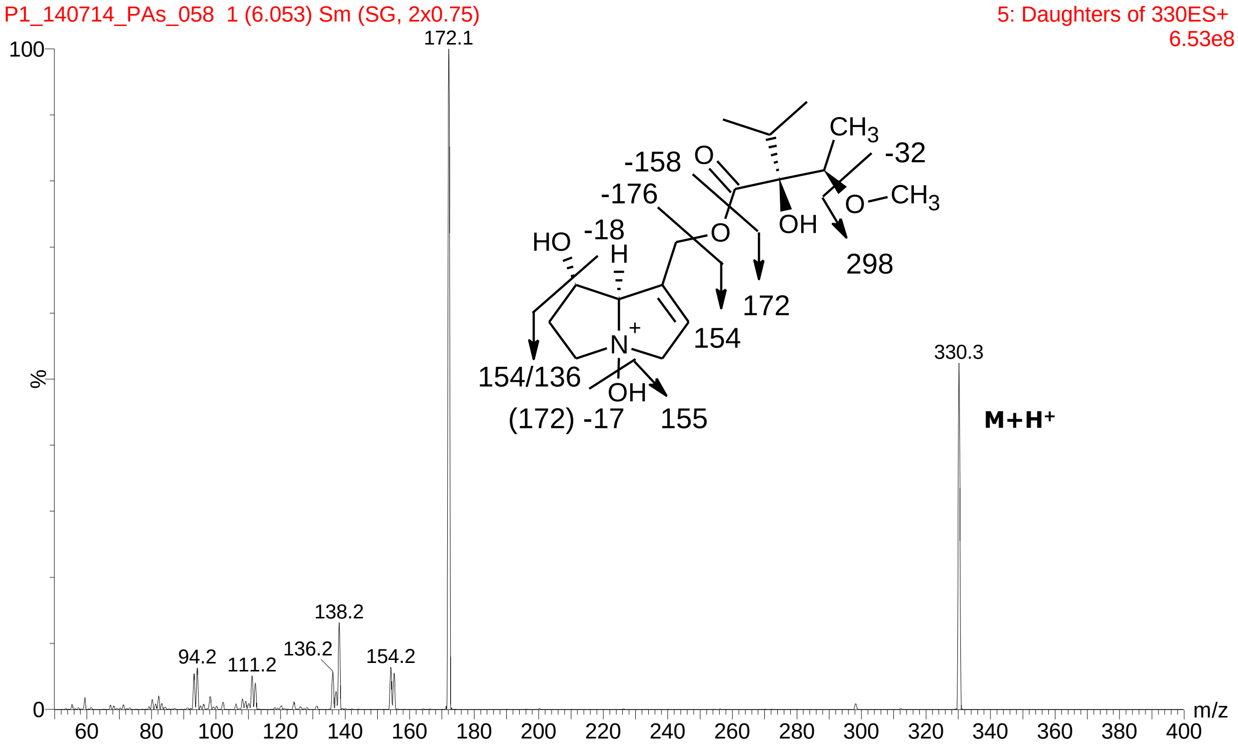

MS/MS fragmentation spectrum of europine (8), RT = 6.70 min

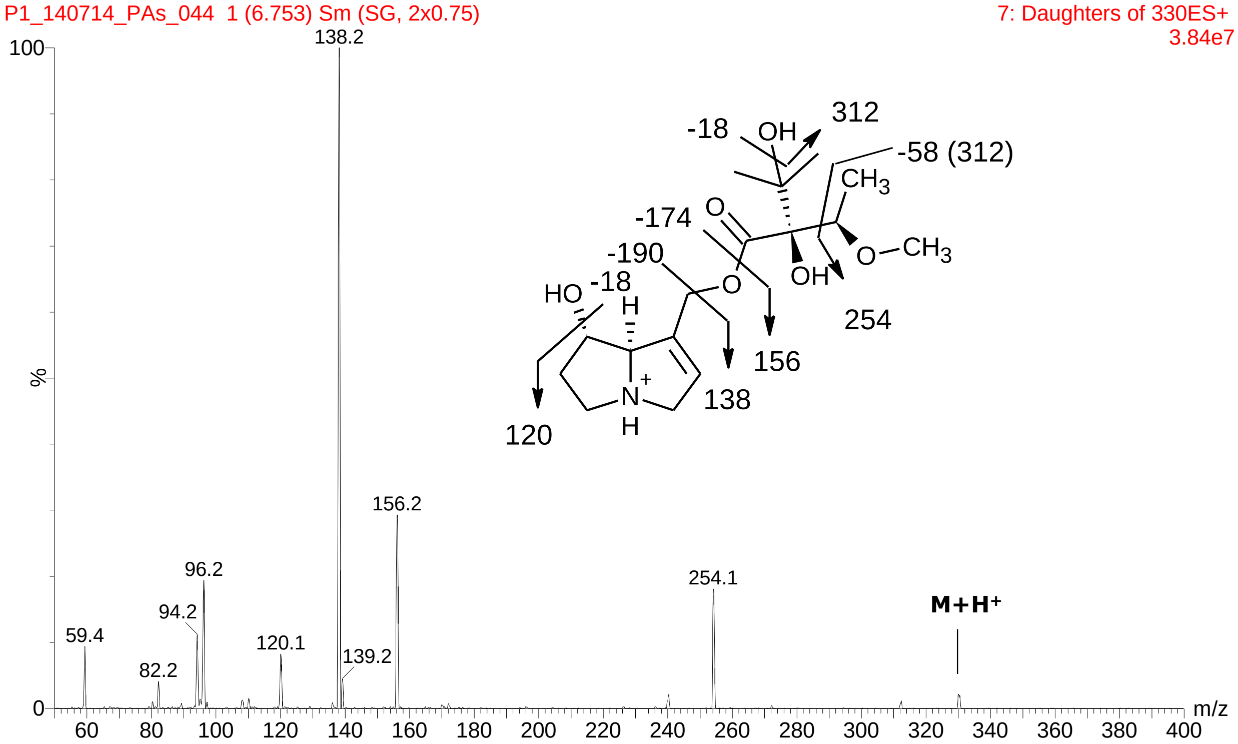

MS/MS fragmentation spectrum of europine-N-oxide (8NO), RT = 4.80 min

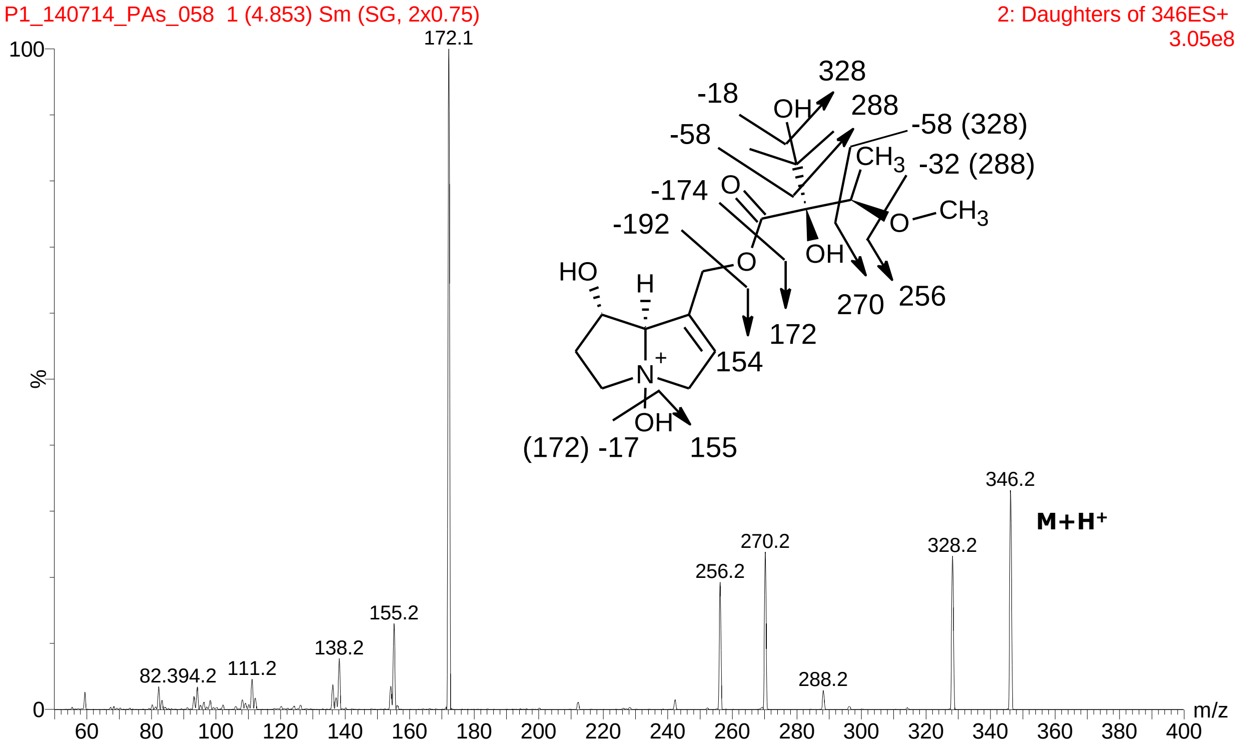

MS/MS fragmentation spectrum of 5′-acetyleuropine (9), RT = 8.20 min

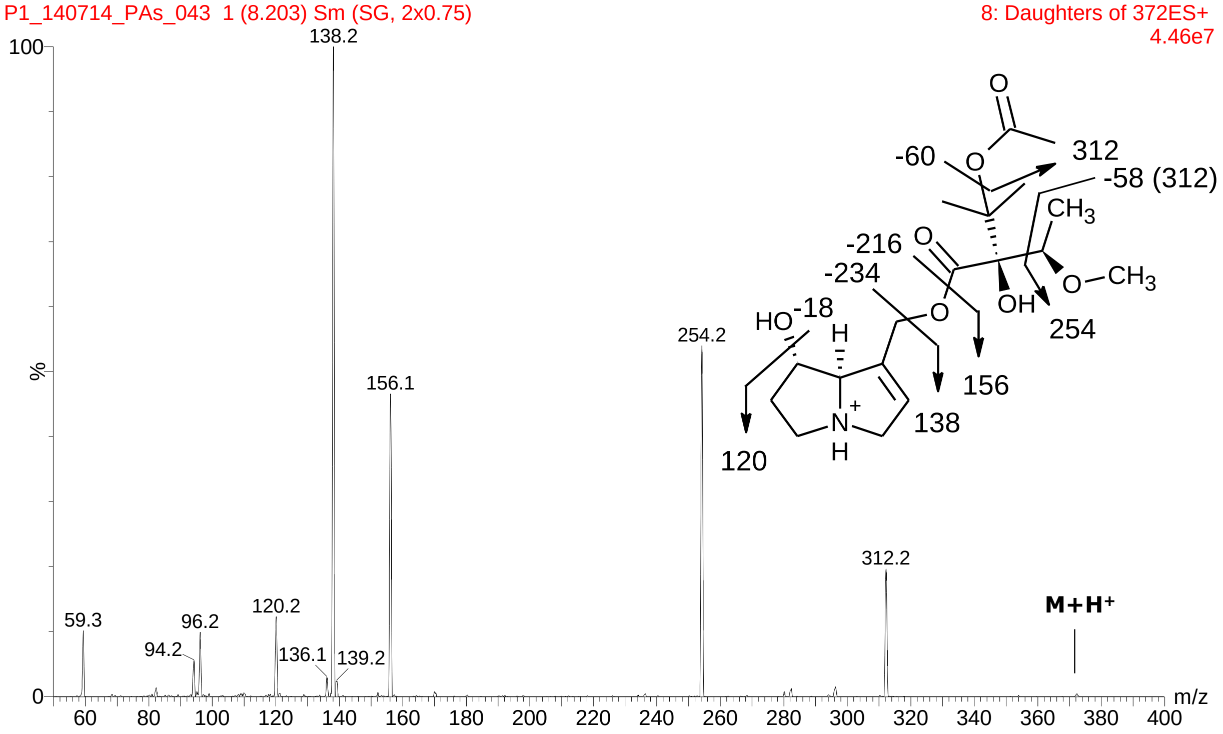

MS/MS fragmentation spectrum of 5'-acetyleuropine-N-oxide (9NO), RT = 6.20 min

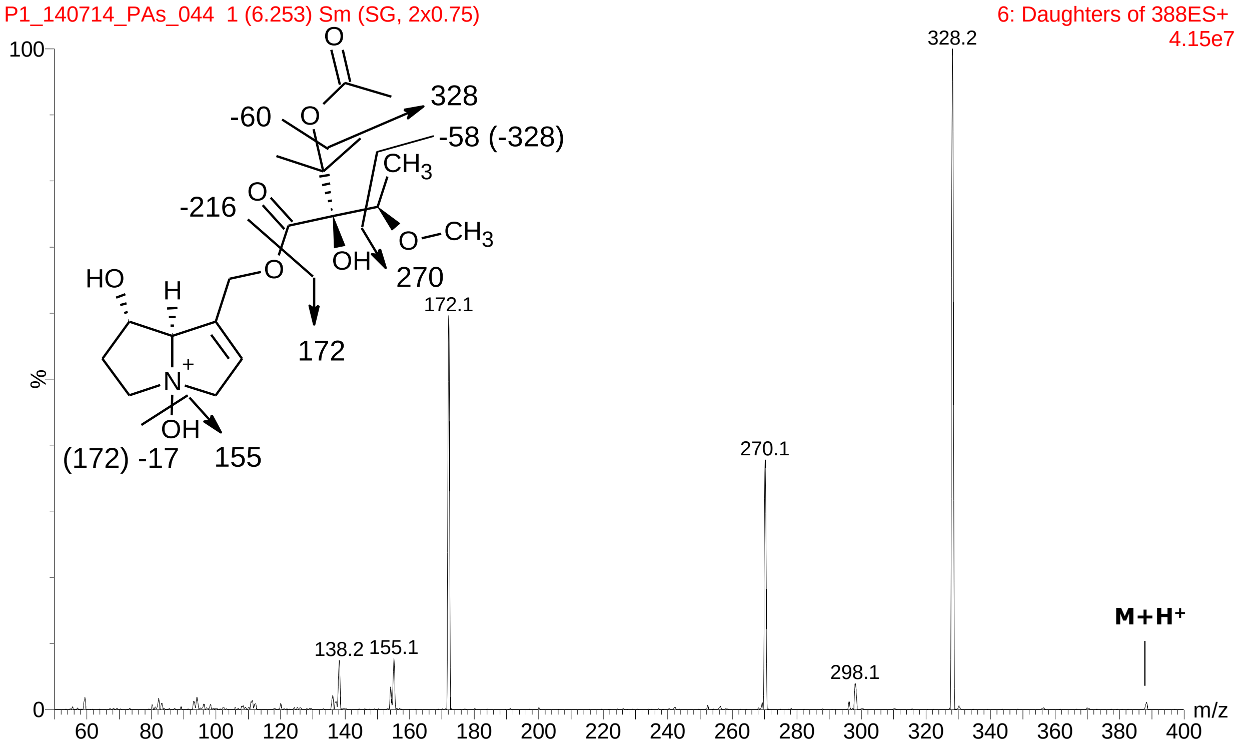

MS/MS fragmentation spectrum of heliosupine (10), RT = 9.75 min

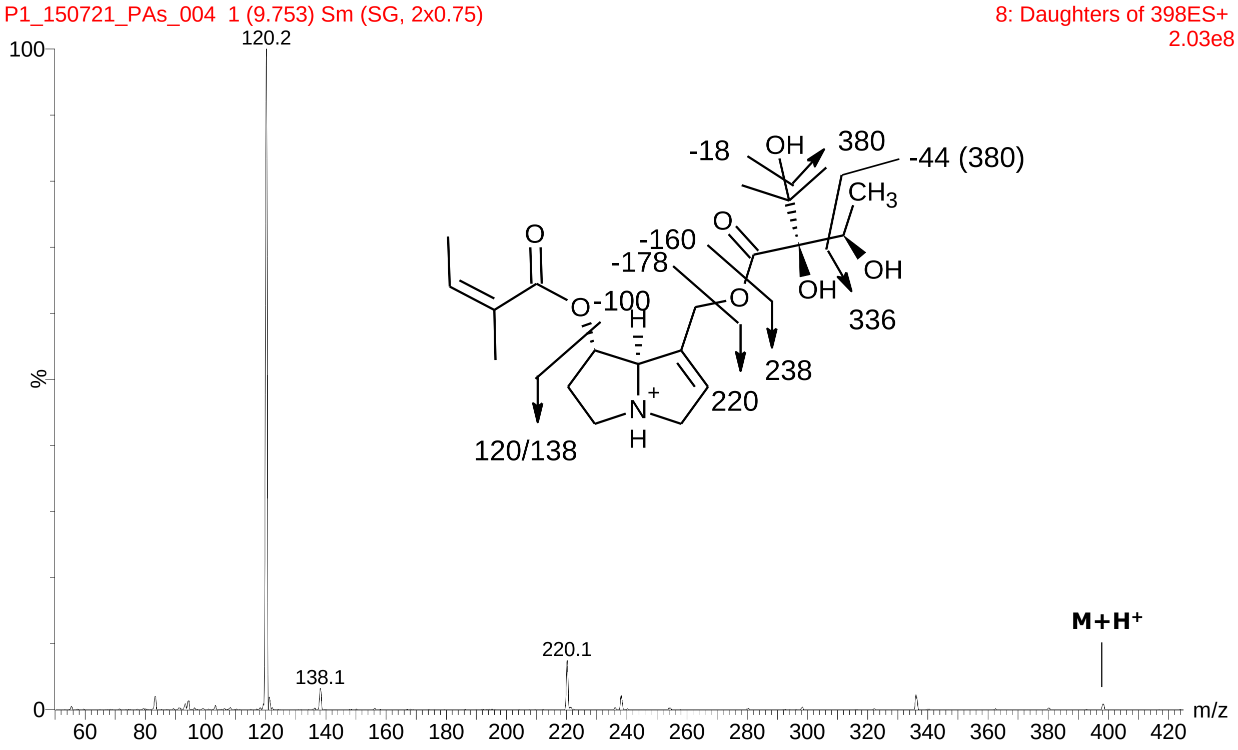

MS/MS fragmentation spectrum of heliosupine-N-oxide (10NO), RT = 7.10 min

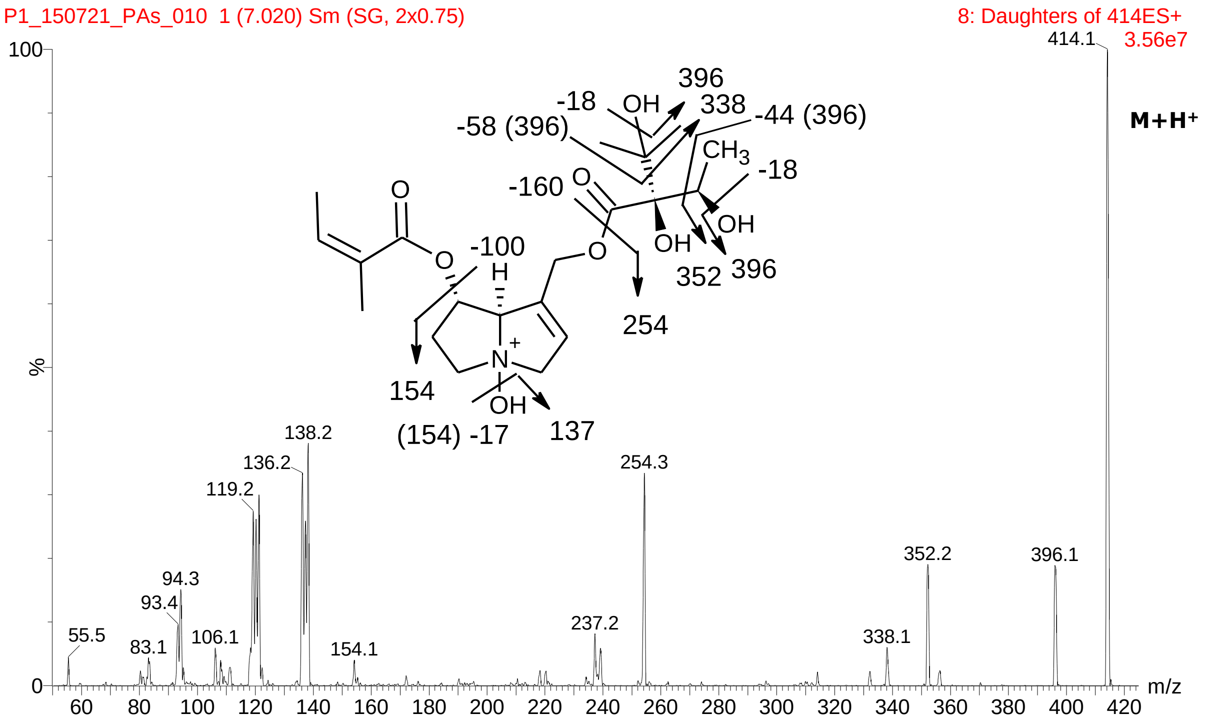

MS/MS fragmentation spectrum of 3'-acetylheliosupine (11), RT =11.00 min

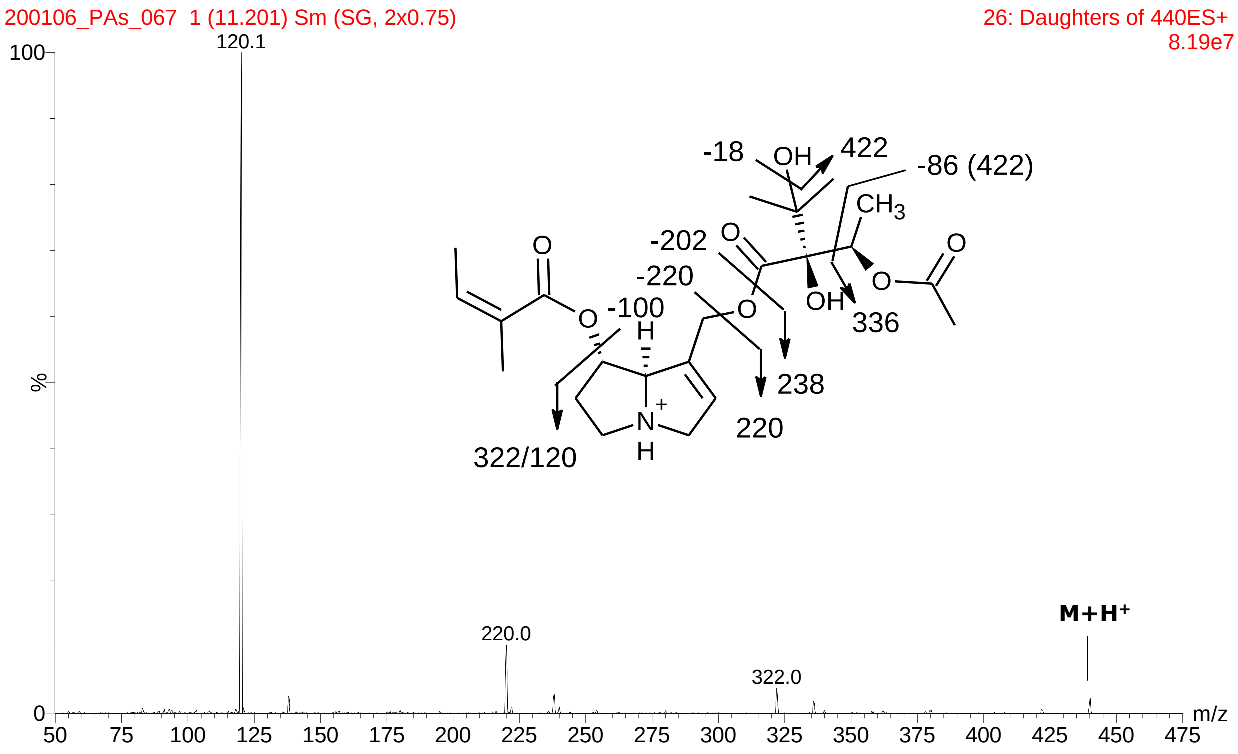

MS/MS fragmentation spectrum of 3'-acetylheliosupine-N-oxide (11NO), RT =7.95 min

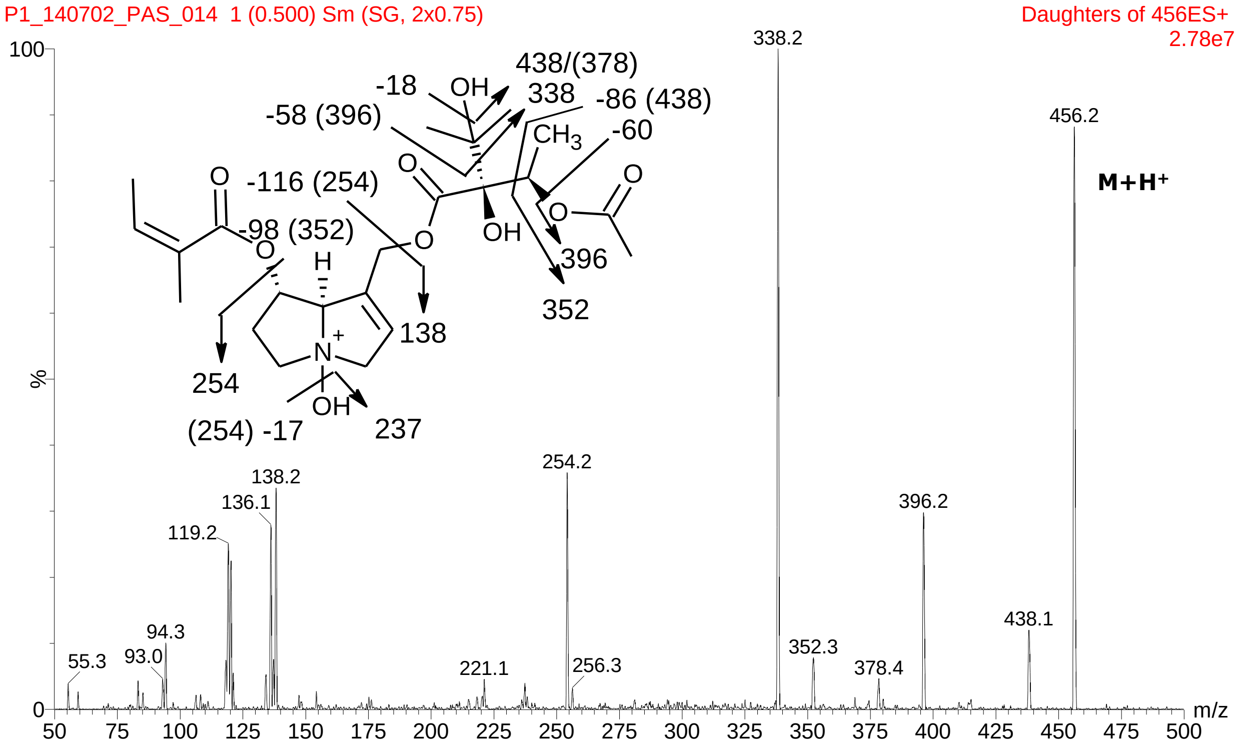

MS/MS fragmentation spectrum of 7-angeloylheliotrine-N-oxide (12NO), RT = 9.30 min

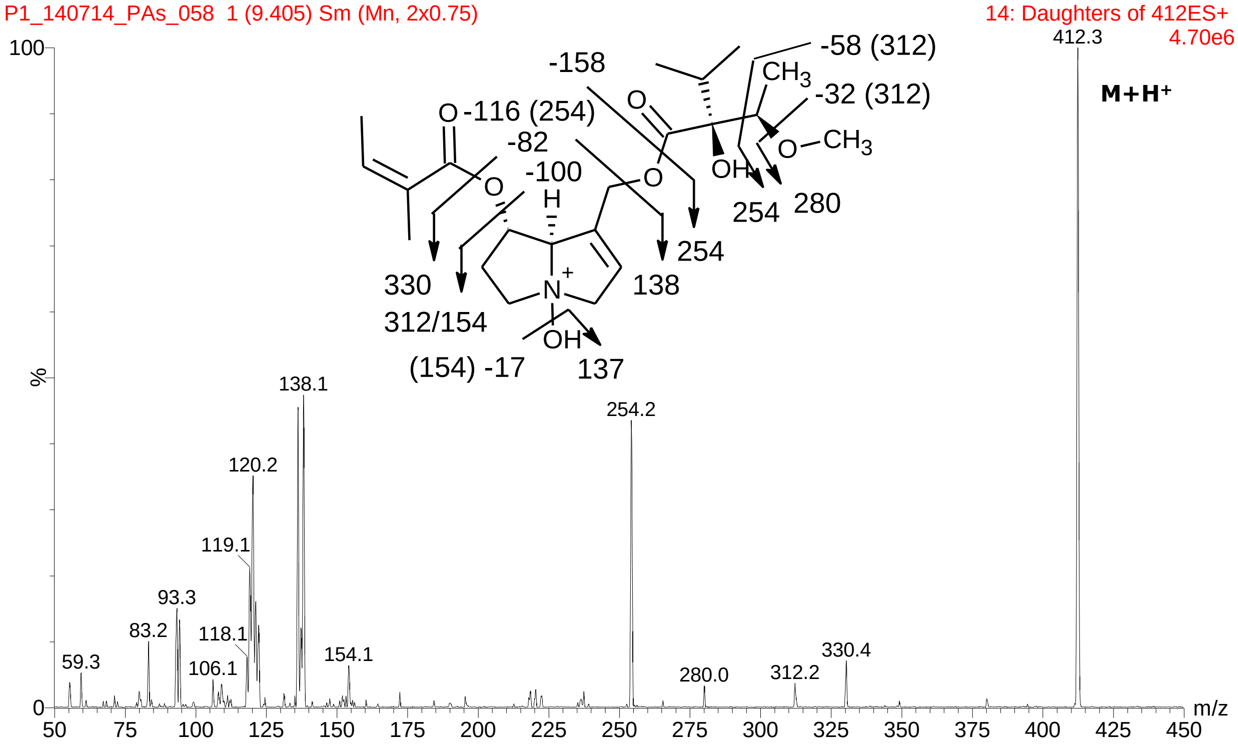

MS/MS fragmentation spectrum of lasiocarpine (13), RT =11.10 min

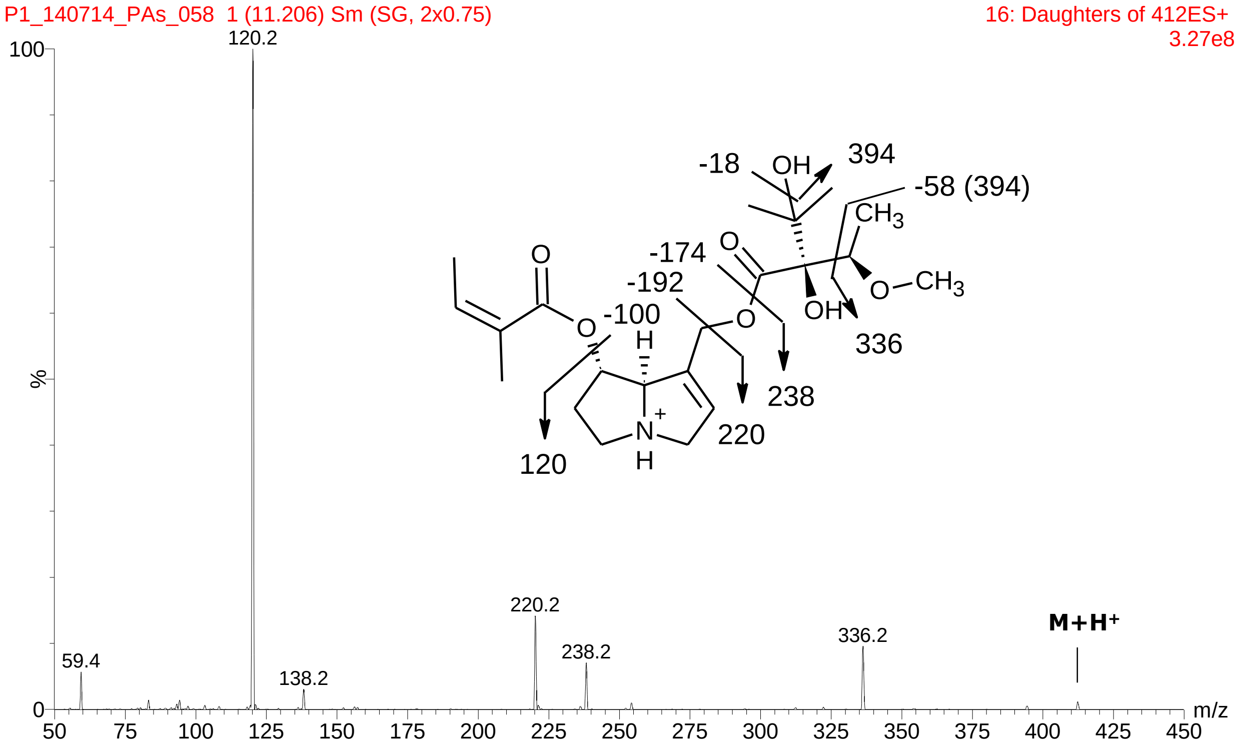

MS/MS fragmentation spectrum of lasiocarpine-N-oxide (13NO), RT =7.90 min

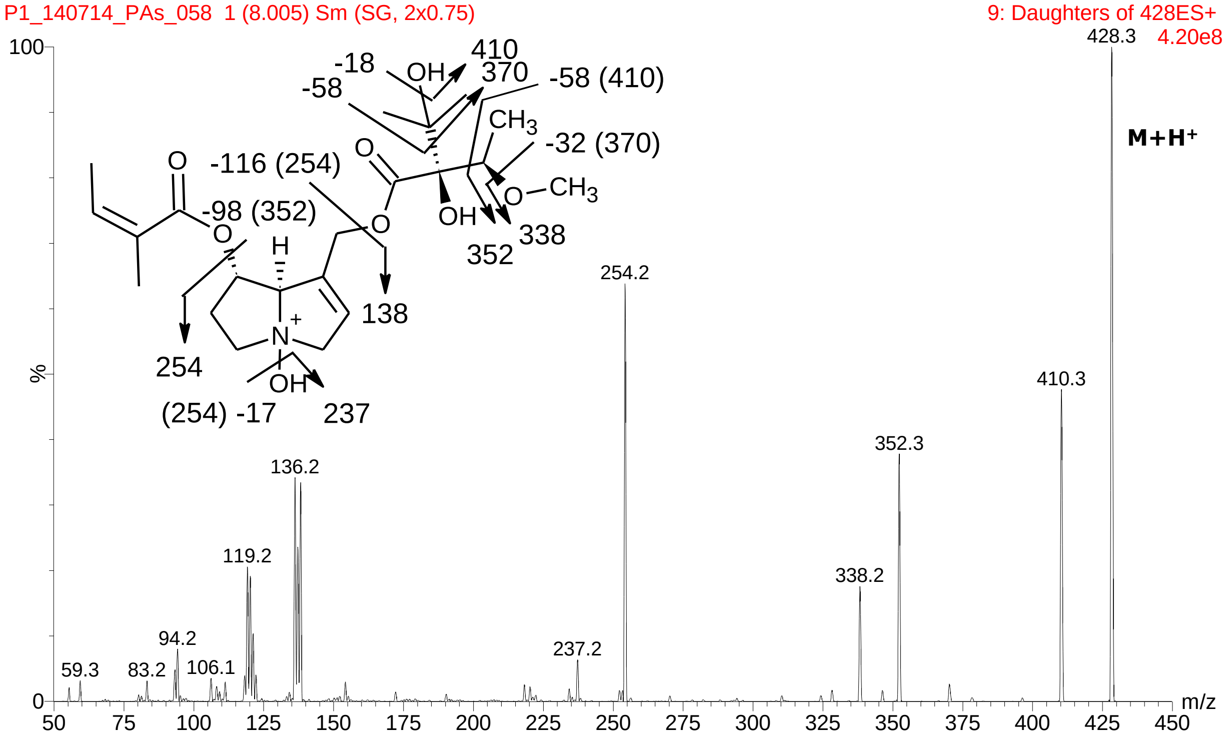

MS/MS fragmentation spectrum of 5'-acetyllasiocarpine (14), RT =12.55 min

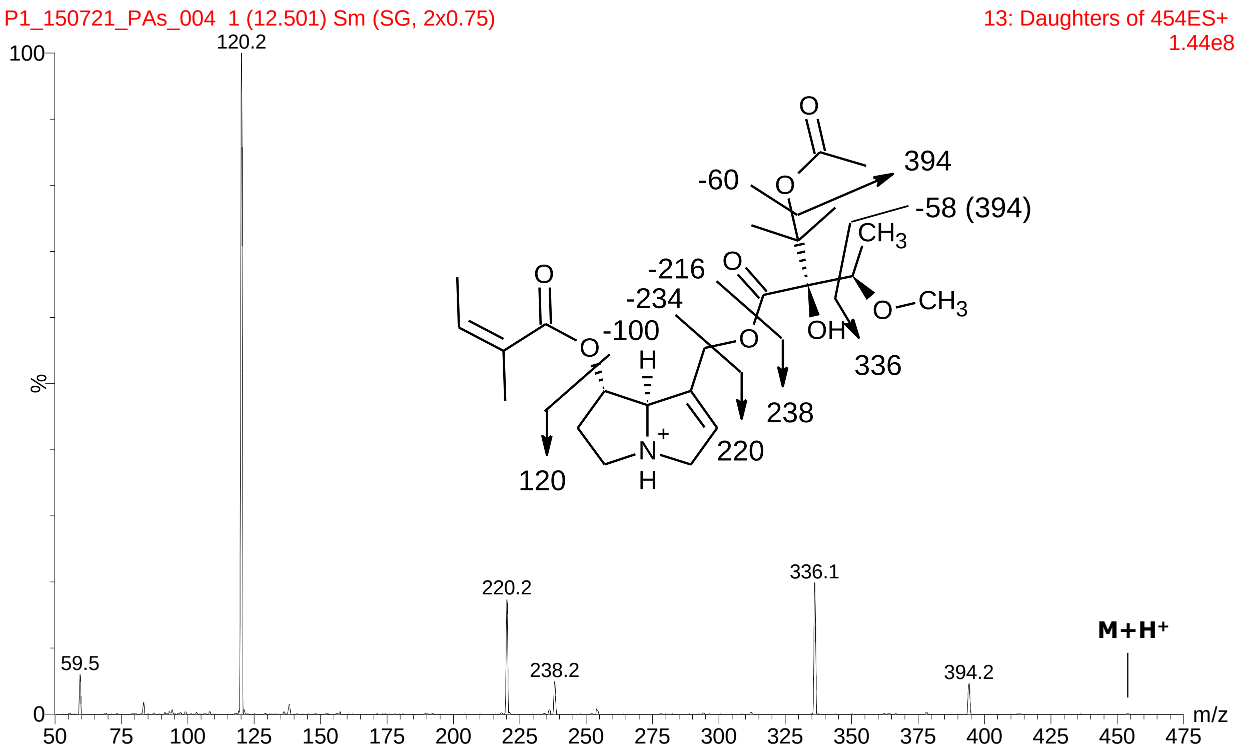

MS/MS fragmentation spectrum of 5'-acetyllasiocarpine-N-oxide (14NO), RT = 9.20 min

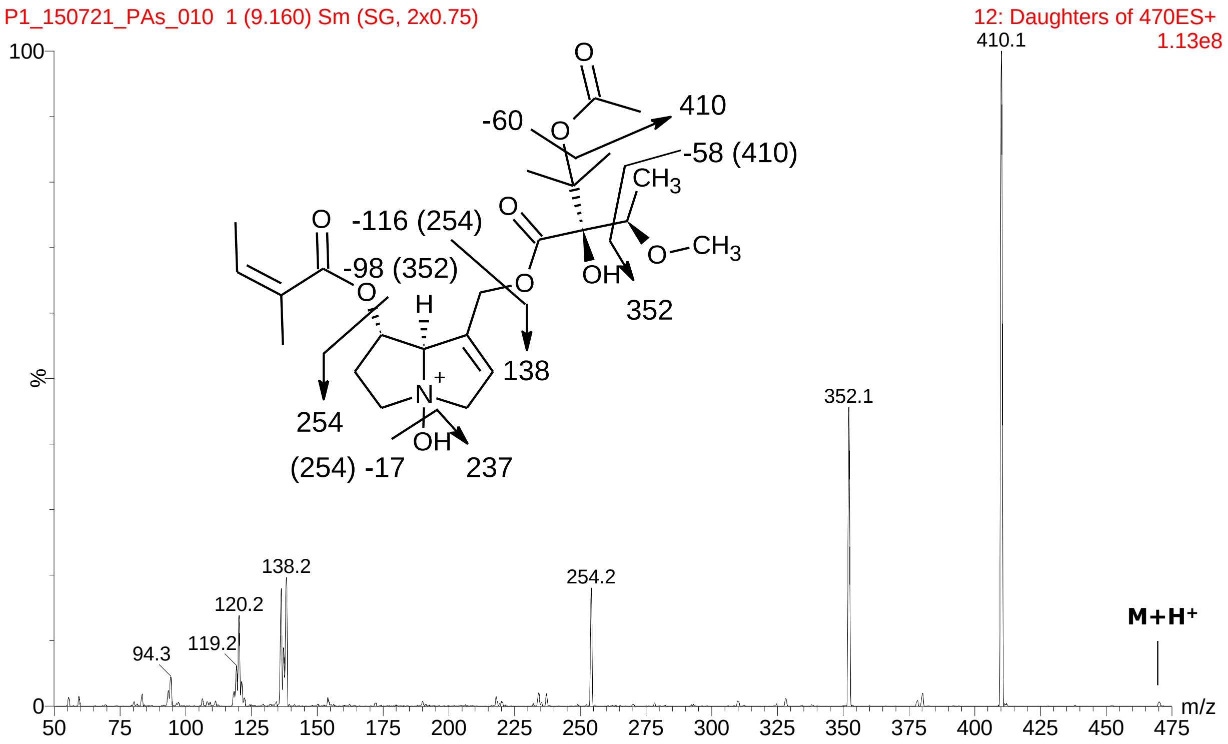

MS/MS fragmentation spectrum of 7-tigloyleuropine (15), RT =10.95 min

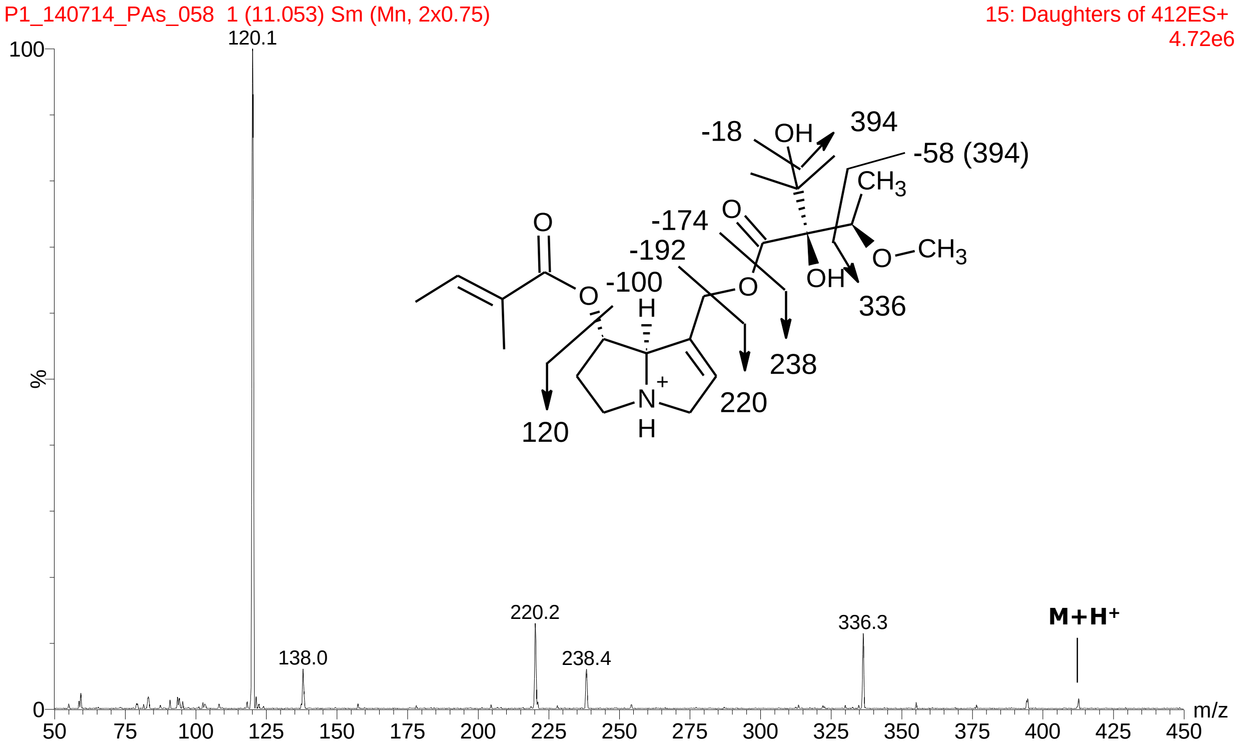

MS/MS fragmentation spectrum of 7-tigloyleuropine-N-oxide (15NO), RT =7.75 min

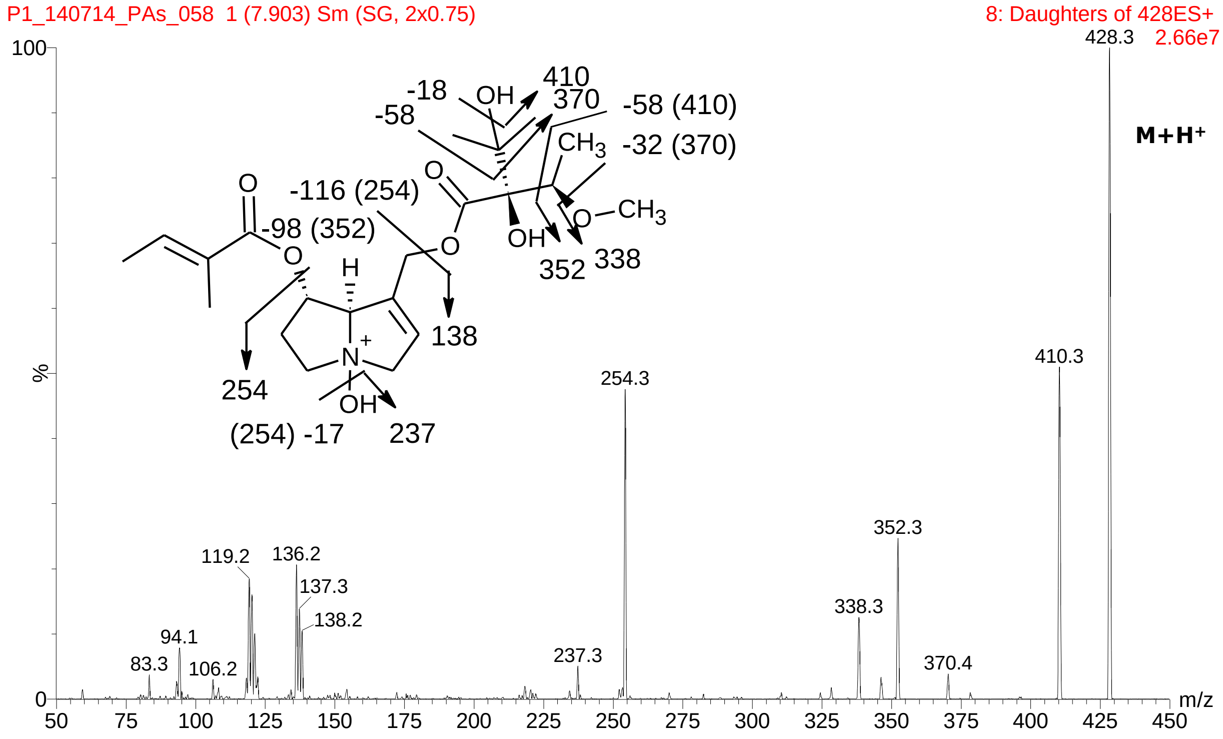

MS/MS fragmentation spectrum of 5'-acetyl-7-tigloyleuropine (16), RT =12.40 min

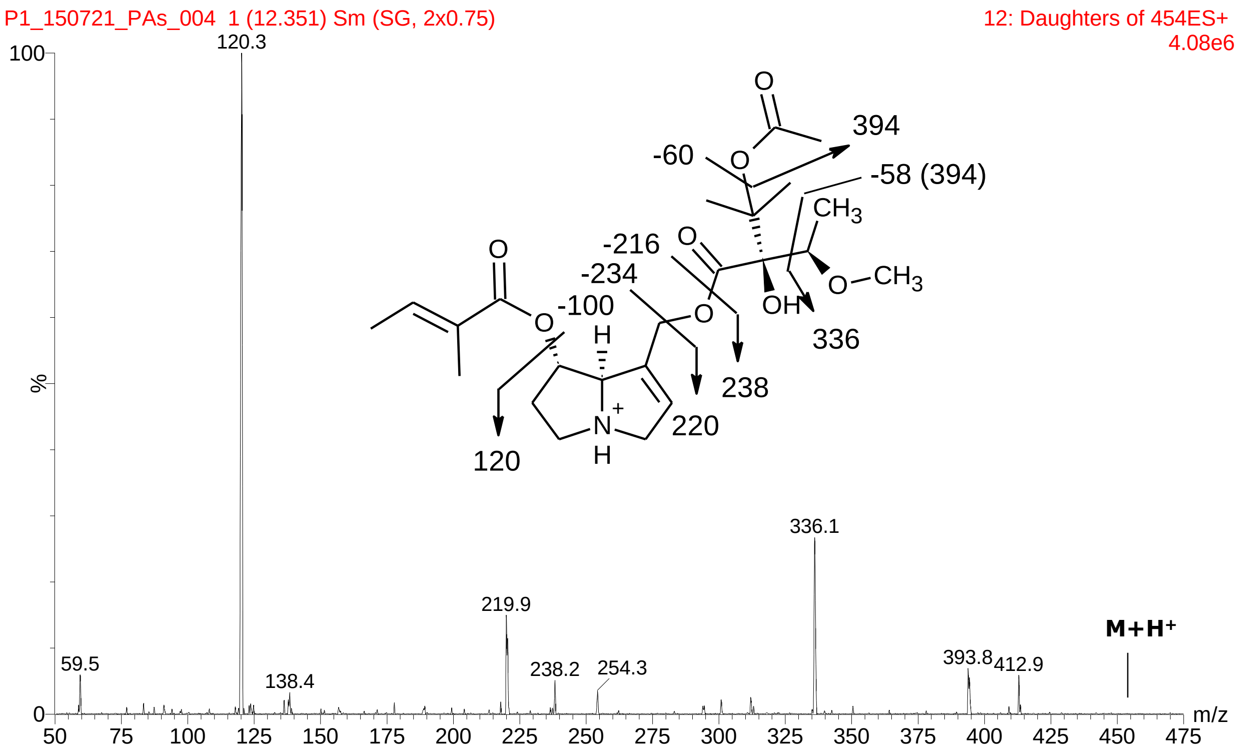

MS/MS fragmentation spectrum of 5'-acetyl-7-tigloyleuropine-N-oxide (16NO), RT = 9.00 min

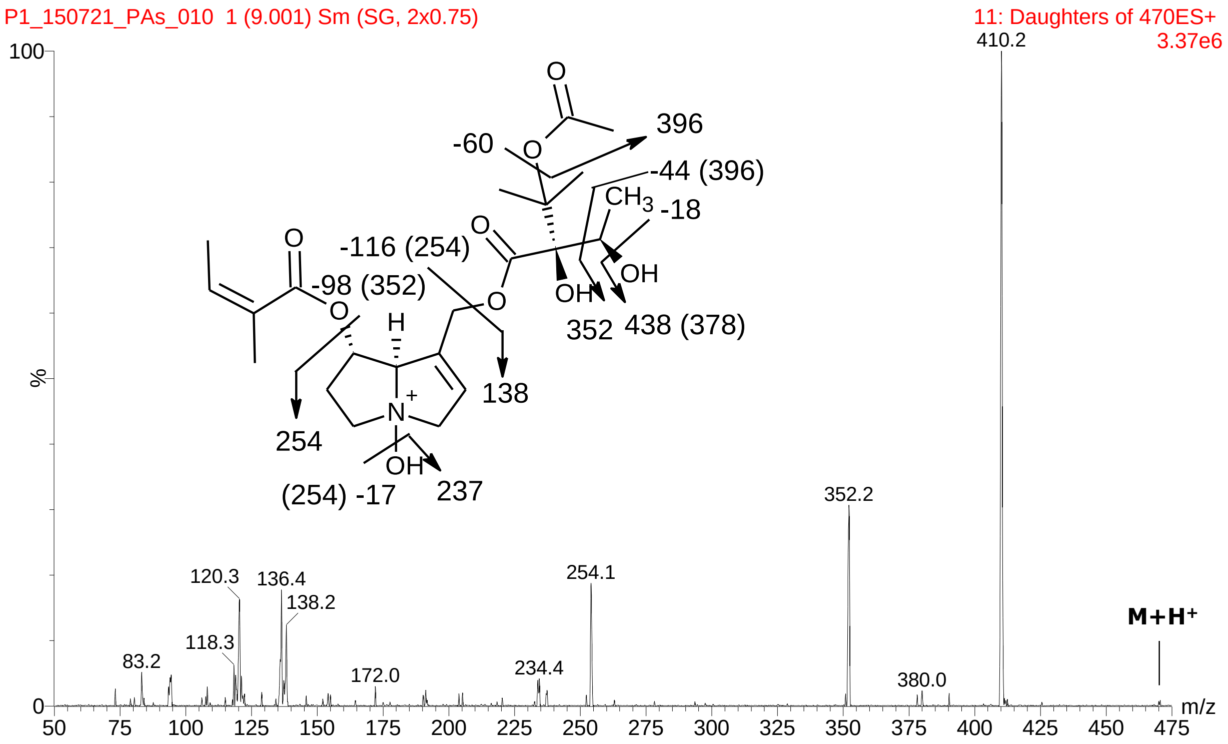

Table 4. Relative PA abundance (%) present in the major plant parts of *H. Europaeum*, *H. rotundifolium* and *H. suaveolens*.[#]

	H. europaeum (H.E)[b]				*H. rotundifolium* (H.R)[b]				*H. suaveolens* (H.S)[b]				
	root	*stem*	*leaf*	*flower*	*root*	*stem*	*leaf*	*flower*	*root*	*stem*	*leaf*	*flower*	**Statistical analysis**
Supinidine-type PAs													
Supinine	0.0 ± 0.0	0.02 ± 0.01	0.04 ± 0.03	0.03 ± 0.02	0.0 ± 0.0	0.0 ± 0.0	0.0 ± 0.0	0.09 ± 0.02	0.0 ± 0.0	0.02 ± 0.03	0.05 ± 0.05	0.05 ± 0.03	NS
Supinine-*N*-oxide	0.02 ± 0.02	0.03 ± 0.03	0.2 ± 0.1	0.20 ± 0.04	0.19 ± 0.03	0.4 ± 0.5	0.1 ± 0.1	1.7 ± 0.3	0.01 ± 0.01	0.1 ± 0.2	0.1 ± 0.1	0.5 ± 0.2	Flowers: species differ from each other at $p < 0.001$
Heleurine	0.03 ± 0.02	0.04 ± 0.08	0.06 ± 0.05	0.08 ± 0.03	0.03 ± 0.04	0.02 ± 0.02	0.02 ± 0.03	0.03 ± 0.01	0.01 ± 0.01	0.03 ± 0.03	0.2 ± 0.3	0.09 ± 0.07	NS
Heleurine-*N*-oxide	0.6 ± 0.4	0.6 ± 0.2	0.9 ± 0.5	1.5 ± 0.7	0.7 ± 0.8	0.3 ± 0.0	0.1 ± 0.2	0.4 ± 0.1	0.5 ± 0.6	1.5 ± 0.7	3.0 ± 3.9	1.7 ± 1.3	Leaves: H.S differ from H.R and H.E at $p < 0.001$ and 0.01, resp.
Heliotrine-type PAs													
Echinatine	0.0 ± 0.0	0.0 ± 0.0	0.0 ± 0.0	0.0 ± 0.0	0.0 ± 0.0	0.0 ± 0.0	0.0 ± 0.0	0.0 ± 0.0	0.0 ± 0.0	0.01 ± 0.01	0.0 ± 0.0	0.0 ± 0.0	NS
Echinatine-*N*-oxide	0.2 ± 0.1	0.2 ± 0.1	0.1 ± 0.1	0.2 ± 0.2	0.01 ± 0.02	0.06 ± 0.01	0.03 ± 0.05	0.05 ± 0.01	0.4 ± 0.4	0.4 ± 0.4	0.2 ± 0.1	0.3 ± 0.2	NS
Heliotrine	1.9 ± 0.6	1.8 ± 0.7	1.4 ± 0.7	1.9 ± 1.1	0.4 ± 0.0	0.19 ± 0.02	0.13 ± 0.01	0.27 ± 0.01	0.9 ±0.2	0.5 ± 0.3	0.3 ± 0.3	0.8 ± 0.6	NS
Heliotrine-*N*-oxide	51.5 ± 10.2	40.0 ± 7.6	45.3 ± 7.8	38.2 ± 19.3	13.8 ± 1.4	5.8 ± 0.9	3.0 ± 1.5	7.3 ± 1.9	29.4 ± 10.0	25.5 ± 14.8	21.9 ± 9.4	15.8 ± 6.1	NS
Europine	0.9 ± 0.7	1.5 ± 0.9	1.1 ± 1.6	1.84 ± 0.9	1.7 ± 0.2	2.5 ± 2.0	1.3 ± 0.3	3.7 ± 0.8	1.1 ± 0.5	1.3 ± 0.7	1.2 ± 0.4	2.8 ± 1.2	NS
Europine-*N*-oxide	22.0 ± 14.1	34.4 ± 10.8	24.1 ± 13.1	40.3 ± 24.6	60.7 ± 9.9	87.2 ± 2.5	93.8 ± 2.3	83.4 ± 4.8	43.4 ± 16.5	57.0 ± 23.1	45.1 ± 14.1	57.0 ± 10.4	Species differ in all plant parts from each other at $p < 0.01$
5'-Acetyleuropine	0.02 ± 0.00	0.1 ± 0.1	0.1 ± 0.1	0.03 ± 0.00	0.1 ± 0.1	0.0 ± 0.0	0.0 ± 0.0	0.01 ± 0.00	0.03 ± 0.01	0.04 ± 0.02	0.04 ± 0.04	0.03 ± 0.02	NS
5'-Acetyleuropine-*N*-oxide	1.3 ± 0.6	3.5 ± 0.9	1.9 ± 1.4	2.0 ± 0.4	3.8 ± 4.5	0.4 ± 0.3	0.3 ± 0.1	0.4 ± 0.01	0.9 ± 0.7	2.5 ± 2.0	2.0 ± 1.6	1.9 ± 1.1	Root and stem: H.R differ from H.E and H.S at $p < 0.01$
Rinderine	0.01 ± 0.00	0.0 ± 0.0	0.01 ± 0.00	0.02 ± 0.00	0.0 ± 0.0	0.0 ± 0.0	0.0 ± 0.0	0.01 ± 0.00	0.0 ± 0.0	0.0 ± 0.0	0.0 ± 0.0	0.1 ± 0.1	NS
Rinderine-*N*-oxide	0.8 ± 0.4	0.3 ± 0.3	1.0 ± 0.5	0.8 ± 0.4	0.5 ± 0.2	0.1 ± 0.1	0.20 ± 0.01	1.0 ± 1.2	0.4 ± 0.3	1.3 ± 1.4	0.7 ± 0.4	5.0 ± 7.5	Flowers: H.S. differ from H.E and H.R at $p < 0.01$
5'-Hydroxyrinder-ine	0.03 ± 0.00	0.0 ± 0.0	0.02 ± 0.00	0.01 ± 0.00	0.2 ± 0.1	0.03 ± 0.01	0.03 ± 0.01	0.02 ± 0.01	0.01 ± 0.02	0.01 ± 0.01	0.1 ± 0.1	0.02 ± 0.02	Roots: H.R differ from H.E and H.S at $p < 0.001$ Leaves: H.S differ from H.R and H.E at $p < 0.01$ and 0.001, resp.
5'-Hydroxyrinder-ine-*N*-oxide	1.5 ± 1.2	0.6 ± 0.2	0.4 ± 0.2	0.5 ± 0.5	9.1 ± 4.4	1.4 ± 1.6	0.8 ± 0.6	0.9 ± 1.1	1.9 ± 0.6	1.2 ± 0.5	1.2 ± 0.6	1.0 ± 0.4	Roots: H.R differ from H.S and H.E at $p < 0.001$
3'-Acetylrinderine	0.01 ± 0.00	0.0 ± 0.0	0.0 ± 0.0	0.0 ± 0.0	0.0 ± 0.0	0.0 ± 0.0	0.0 ± 0.0	0.0 ± 0.0	0.0 ± 0.0	0.0 ± 0.0	0.0 ± 0.0	0.0 ± 0.0	NS

3'-Acetylrinderine-*N*-oxide	0.04 ± 0.10	0.0 ± 0.0	0.0 ± 0.0	0.03 ± 0.1	0.2 ± 0.2	015 ± 0.1	0.01 ± 0.02	0.05 ± 0.07	0.05 ± 0.05	0.04 ± 0.04	0.0 ± 0.0	0.1 ± 0.1	NS
Lasiocarpine-type PAs													
7-Angeloylheliotrine-*N*-oxide	0.08 ± 0.1	0.01 ± 0.00	0.02 ± 0.00	0.03 ± 0.03	0.0 ± 0.0	0.0 ± 0.0	0.0 ± 0.0	0.0 ± 0.0	0.0 ± 0.0	0.0 ± 0.0	0.0 ± 0.0	0.0 ± 0.0	NS
Heliosupine	0.2 ± 0.3	0.02 ± 0.00	0.03 ± 0.00	0.01 ± 0.00	0.21 ± 0.01	0.0 ± 0.0	0.0 ± 0.0	0.0 ± 0.0	0.3 ± 0.2	0.03 ±0.04	0.0 ± 0.0	0.01 ± 0.01	NS
Heliosupine-*N*-oxide	1.0 ± 0.9	0.5 ± 0.4	0.4 ± 0.5	0.2 ± 0.1	0.9 ± 0.7	0.04 ± 0.02	0.0 ± 0.0	0.01 ± 0.01	1.2 ± 1.4	0.5 ± 0.5	0.6 ± 0.7	0.3 ± 0.3	NS
3'-Acetylheliosupine	0.3 ± 0.4	0.03 ± 0.00	0.0 ± 0.0	0.0 ± 0.0	0.10 ± 0.00	0.0 ± 0.0	0.0 ± 0.0	0.0 ± 0.0	0.3 ± 0.4	0.03 ± 0.05	0.01 ± 0.01	0.00 ± 0.01	NS
3'-Acetylheliosupine-*N*-oxide	1.3 ± 1.4	0.2 ± 0.5	0.2 ± 0.3	0.2 ± 0.3	1.7 ± 2.4	0.1 ± 0.1	0.0 ± 0.0	0.01 ± 0.01	1.4 ± 1.8	0.6 ± 0.9	0.3 ± 0.5	0.4 ±0.6	NS
Lasiocarpine	1.6 ± 2.2	0.6 ± 0.1	0.6 ± 0.3	0.3 ± 0.1	0.3 ± 0.1	0.01 ± 0.02	0.00 ± 0.01	0.04 ± 0.03	2.9 ± 2.8	0.3 ± 0.2	0.5 ± 0.3	0.33 ± 0.3	Roots: all species differ from each other at $p < 0.01$
Lasiocarpine-*N*-oxide	14.2 ± 8.3	11.5 ± 5.1	17.8 ± 9.7	10.3 ± 6.4	5.4 ± 4.5	1.2 ± 1.6	0.1 ± 0.1	0.6 ± 0.7	14.2 ± 5.6	5.4 ± 4.3	18.5 ± 19.3	10.9 ± 12.1	NS
5'-Acetyllasiocarpine	0.03 ± 0.00	0.1 ± 0.1	0.1 ± 0.2	0.03 ± 0.00	0.0 ± 0.0	0.0 ± 0.0	0.0 ± 0.0	0.0 ± 0.0	0.04 ± 0.04	0.02 ± 0.01	0.06 ± 0.05	0.03 ± 0.02	NS
5'-Acetyllasiocarpine-*N*-oxide	0.4 ± 0.4	2.3 ± 1.5	2.9 ± 3.5	1.2 ± 0.9	0.2 ± 0.1	0.1 ± 0.2	0.02 ± 0.03	0.04 ± 0.01	0.5 ± 0.5	0.9 ± 0.7	2.0 ± 2.1	0.9 ± 0.8	Stem: H.R and H.E differ at $p < 0.01$ Leaves: H.R differ from H.E and H.S at $p < 0.001$ and 0.01, resp.
7-Tigloyleuropine	0.01 ± 0.01	0.04 ± 0.1	0.05 ± 0.04	0.01 ± 0.00	0.0 ± 0.0	0.01 ± 0.00	0.0 ± 0.0	0.0 ± 0.0	0.03 ± 0.02	0.02 ± 0.01	0.05 ± 0.02	0.01 ± 0.01	NS
7-Tigloyleuropine-*N*-oxide	0.05 ± 0.01	1.4 ± 0.7	1.3 ± 0.6	0.2 ± 0.2	0.1 ± 0.1	0.1 ± 0.1	0.01 ± 0.02	0.0 ± 0.0	0.12 ± 0.07	0.8 ± 0.9	1.8 ± 2.2	0.1 ± 0.1	Stem: H.R. and H.E differ at $p < 0.001$ Leaves: H.R differ from H.E and H.S at $p < 0.01$ and $p < 0.001$, resp.
5'-Acetyl-7-Tigloyleuropine	0.0 ± 0.0	0.0 ± 0.0	0.01 ± 0.00	0.0 ± 0.0	0.0 ± 0.0	0.0 ± 0.0	0.0 ± 0.0	0.0 ± 0.0	0.0 ± 0.0	0.01 ± 0.00	0.0 ± 0.0	0.0 ± 0.0	NS
5'-Acetyl-7-Tigloyleuropine--*N*-oxide	0.0 ± 0.0	0.2 ± 0.1	0.3 ± 0.2	0.02 ± 0.01	0.0 ± 0.0	0.0 ± 0.0	0.0 ± 0.0	0.0 ± 0.0	0.01 ± 0.01	0.1 ± 0.2	0.2 ± 0.2	0.01 ± 0.01	Stem: H.R differ from H.E and H.S at $p < 0.001$ and 0.05, resp. Leaves: H.R differ from H.E and H.S at $p < 0.001$

[#]Mean PA concentrations of the same plant parts (3-5 replicates for each organ part of each species) of each *Heliotropium* species were compared to each other for statistical significance by 2-way ANOVA followed by Bonferroni multiple comparison test
